# Gallic acid alleviates exercise-induced muscle damage by inhibiting mitochondrial oxidative stress and ferroptosis

**DOI:** 10.1186/s12967-024-06042-5

**Published:** 2025-01-08

**Authors:** Likai Yu, Di Tian, Zishan Su, Li Zhang, Shaobo Guo, Wenhui Zhu, Yuan Fang, Peimin Wang, Nongshan Zhang

**Affiliations:** 1 Department of Orthopedics, Affiliated Hospital of Nanjing University of Chinese Medicine / Jiangsu Province Hospital of Chinese Medicine, Nanjing, Jiangsu 210029 China; 2 Key Laboratory for Metabolic Diseases in Chinese Medicine, First College of Clinical Medicine, Nanjing University of Chinese Medicine, Nanjing, Jiangsu 210023 China; 3 Orthopedics of Traditional Chinese Medicine, Zhongda Hospital Southeast University, Nanjing, Jiangsu 210009 China

**Keywords:** Gallic acid, Excessive exercise, Skeletal muscle injury, Mitochondrial oxidative stress, Ferroptosis

## Abstract

**Background:**

Skeletal muscle injury caused by excessive exercise is one of the most commonly seen clinical diseases. It is indispensable to explore drugs for treating and relieving skeletal muscle injury. Gallic acid (GA) is a polyphenolic extract that has anti-inflammatory and antioxidant biological activities. However, its function and mechanism in skeletal muscle injury remain unclear.

**Methods:**

We first established a skeletal muscle injury model caused by excessive exercise. Histopathological analysis was used to determine the severity of skeletal muscle injury in mice. Techniques such as ELISA, Western blot, and RT-qPCR were used to measure skeletal muscle injury markers including CK, LDH, IL-6, TNF-α, and ferroptosis-related indicators such as Fe^2+^, MDA, COX2, and GPX4. Transmission electron microscopy was used to observe the morphology of mitochondria. JC-1, DHE, and C11-BODIPY 581/591 probes were used to detect mitochondrial membrane potential, mitochondrial reactive oxygen species (mtROS), and lipid peroxidation levels.

**Results:**

The results of this study indicate that GA has a positive therapeutic effect on skeletal muscle inflammation and injury induced by excessive exercise. On the one hand, GA can alleviate skeletal muscle mitochondrial injury and redox imbalance by reducing mitochondrial membrane potential level and increasing ATP production. On the other hand, GA can inhibit ferroptosis in skeletal muscle cells induced by excessive exercise through its antioxidant and anti-iron accumulation ability.

**Conclusions:**

In summary, GA protects against skeletal muscle injury induced by excessive exercise by inhibiting mitochondrial oxidative stress and ferroptosis pathways, providing new evidence for GA as a promising therapeutic agent for skeletal muscle injury.

**Supplementary Information:**

The online version contains supplementary material available at 10.1186/s12967-024-06042-5.

## Introduction

The human body is susceptible to exercise-induced muscle damage (EIMD) after undergoing loaded exercise, especially high-intensity centrifugal contractions [1]. This reaction can lead to various symptoms such as decreased muscle strength, limited range of motion, and delayed-onset muscle soreness, which can negatively affect an individual’s ability to exercise and exercise compliance [2]. At the onset of EIMD, muscle proteins are released into the circulation in large quantities, such as creatine kinase (CK) and lactate dehydrogenase (LDH), while inflammatory markers, such as interleukin (IL) and tumour necrosis factor-alpha (TNF-α), are markedly elevated, which is a consequence of the correlation between EIMD and oxidative stress [3]. The relationship between exercise and oxidative stress first appeared in the literature in 1978, and based on this, a growing number of studies have confirmed that all types of strenuous exercise (prolonged endurance exercise, high-intensity anaerobic exercise, etc.) lead to oxidative stress, and increased oxidative biomarkers have been found in skeletal muscle and blood [4]. At the same time, high levels of reactive oxygen species (ROS) expressed in response to stimuli are also negatively affecting skeletal muscle function, e.g. muscle fibres require appropriate levels of ROS to produce maximal isometric force, and treatment with the antioxidant N-acetylcysteine slows the rate at which muscle fatigue occurs with prolonged exercise [5]. Therefore, exploring the interconnecting mechanisms in skeletal muscle that control redox homeostasis and improving its antioxidant capacity is an important research idea to improve human exercise adaptation problems.

Healthy skeletal muscle has some regenerative capacity and can repair damage through a range of mechanisms, such as autophagy and selectively activated M2-type macrophages, but a large accumulation of dead cells is a rate-limiting step in muscle healing and can have deleterious effects on local tissues [6]. Ferroptosis is a form of non-apoptotic cell death dependent on iron and lipid reactive oxygen species clusters; oxidative stress and cellular antioxidant levels are important regulators of lipid peroxidation in ferroptosis, while lipophilic antioxidants (vitamin E, biologically active polyphenols, etc.) can prevent ferroptosis [7]. Glutathione peroxide reductase (GPX4) is a phospholipid hydroperoxide reductase that plays a dominant role in the regulation of ferroptosis, and its production is associated with glutathione (GSH) synthesis [8]. As the most important reductase, silencing and inhibition of GPX4 triggers ferroptosis, whereas overexpression of GPX4 reduces ROS levels and thus prevents cellular ferroptosis [9]. For example, cysteine protease inhibitors can inhibit gastric cancer progression by regulating GPX4 expression and intracellular ROS levels [10]. A high-iron diet increases hepatic iron levels and promotes GSH depletion, lipid peroxidation, and oxidative stress, decreasing GPX4 expression and ultimately activating cellular ferroptosis [11]. Based on the properties of EIMD, high levels of expressed ROS provide the material basis for ferroptosis in skeletal muscle cells, and it has been shown that mitochondrial production of ROS, DNA stress, and metabolic reprogramming are required for the activation of lipid peroxidation and ferroptosis programmes [12]. Meanwhile, several antioxidants targeting mitochondrial ROS (Mito TEMPO et al.) have been shown to inhibit ferroptosis in a wide range of cells, such as cancer cells, cardiomyocytes, and hippocampal neuronal cells [12]. Numerous studies have identified ferroptosis as a potential therapeutic target for skeletal muscle disorders, with effects involving several diseases such as sarcopenia, rhabdomyolysis, and rhabdomyosarcoma [13, 14]. In senescent mice, the absence of transferrin receptor 1 leads to activation of lipid peroxidation and ferroptosis, which further inhibits satellite cell proliferation and impairs skeletal muscle regeneration [15]. Ferroptosis also significantly impedes the differentiation of C2C12 myoblasts into myotubes and affects muscle production [16]. One study found changes in several ferroptosis-related genes, including heme oxygenase-1 and prostaglandin-endoperoxide synthase, in skeletal muscle samples from older individuals with sarcopenia [17]. Notably, it has been reported that excessive exercise in mice leads to abnormal iron accumulation in skeletal muscle, accompanied by increased lipid peroxidation and decreased GPX4 expression. These findings suggest that exercise-induced muscle fatigue and damage may be associated with ferroptosis [17]. Additionally, researchers have found that activation of the Nuclear factor erythroid 2-related factor 2 signaling pathway can inhibit ferroptosis by reducing ROS production and enhancing GPX4 and GSH activity. This activation alleviates skeletal muscle damage and fatigue symptoms induced by intense exercise in mice [18]. Moreover, treatments targeting lipid peroxidation have been shown to inhibit ferroptosis and facilitate skeletal muscle repair following contusion injuries in rats [19]. However, the mechanism of ferroptosis in EMID has not been fully elucidated and remains to be fully investigated.

Gallic acid (GA) is a naturally occurring plant phenolic compound that is widely found in a variety of plants and fruits and has received continued attention for its powerful anti-inflammatory and antioxidant properties [20]. In the hepatitis C virus environment, GA reduces ROS production in cells early in exposure as a means of down-regulating viral replication in hepatocellular carcinoma cells [21]. Similarly, in dust-exposure-induced alcoholic non-fatty liver disease rats, GA can protect the liver from inflammatory response due to the dust stimulation by inhibiting oxidative stress [22]. Mitochondria are the main organelles responsible for ROS generation, whereas GA can target mitochondria-specific signal transduction pathways involved in multiple molecular mechanisms such as ROS generation, mitochondrial respiration, and cell death [23]. It has also been demonstrated that GA can activate the expression of the ferroptosis-related protein GPX4 in HepG2 cells by blocking β-catenin transport in the nucleus, thereby reducing the development of hepatocellular carcinoma [24]. Here, we investigated the potential protective role of GA in skeletal muscle and hypothesised that it improves EIMD through its anti-ferroptosis activity.

We provide new insights into the cytoprotective effects of GA on oxidative stress and ferroptosis in the present study and provide conclusive evidence in a mouse model of EIMD and in skeletal muscle cells. Our findings suggest that GA modulates ferroptosis and attenuates EIMD by activating GPX4 protein, inhibiting iron accumulation, and oxidative stress in skeletal muscle cells and mouse muscle. Therefore, we conclude that GA may help alleviate the symptoms of skeletal muscle injury after strenuous exercise, improve exercise tolerance, and serve as a new option for clinical treatment.

## Materials and methods

### Mouse exercise injury model induced by treadmill exercise

Eight-week old SPF grade male C57BL/6 mice, weighing 20 ± 5 g, were housed at the Experimental Animal Center of Nanjing University of Chinese Medicine. After one week of adaptive feeding, they were randomly divided into eight groups: Control (*n* = 10), Treadmill Exercise (TE) (*n* = 10), Treadmill Exercise with Ferrostatin-1 (TE + Fer-1) (*n* = 10), and Treadmill Exercise with Gallic Acid (TE + GA) (*n* = 10) in the first experiment. In the second experiment, the groups were Control (*n* = 10), TE (*n* = 10), TE + GA (*n* = 10), and Treadmill Exercise with Mitoquinone Mesylate (TE + MitoQ) (*n* = 10). For the treadmill groups, excessive mechanical stress was applied through running. Reference for the establishment of the skeletal muscle injury model was obtained from the literature and was modified [25, 26]. After the adaptive feeding period, a three-day adaptive training was conducted on the treadmill with a speed of 10 m/min, twice daily, for 15 min each time. Following the adaptive training, formal training began, with a treadmill set at a speed of 25 m/min and a slope of 15°, once daily for 1 h, 6 days per week, for 8 weeks. The TE + Fer-1 group received Ferrostatin-1 (i.p. 5 mg/kg/d, MCE, USA) [27] intervention for 4 weeks starting in the 4th week of treadmill exercise. The TE + GA group received Gallic acid (i.g. 200 mg/kg/d, MCE, USA) [28] intervention for 4 weeks starting in the 4th week of treadmill exercise. The TE + MitoQ group received Mitoquinone mesylate (i.p. 5 mg/kg/d, MCE, USA) [29] intervention for 4 weeks starting in the 4th week of treadmill exercise. The control group was maintained on a regular feeding schedule without additional interventions. Mice from each group were euthanized after the treadmill exercise, and their knee joints were dissected and processed. All experiments were conducted in accordance with ethical guidelines, and all protocols were approved by the Experimental Animal Management Committee and the Experimental Animal Ethics Committee of Nanjing University of Chinese Medicine (ethical license number: 202308A002).

### Detection of skeletal muscle injury markers

The concentrations of IL-6 and TNF-α in the skeletal muscle homogenate of mice after modeling were detected using an ELISA kit. The ELISA kit was purchased from mlbio and was used according to the manufacturer’s protocol. The levels of CK or LDH in the skeletal muscle homogenate or primary skeletal muscle cells of mice were measured using the Creatine kinase assay kit (Jianchen, China) and Lactate dehydrogenase assay kit (Jianchen, China), respectively. Specific operations were carried out according to the manufacturer’s instructions.

### Detection of ferroptosis-related markers

The levels of ferrous iron (Fe^2+^) or Malondialdehyde (MDA) in the skeletal muscle homogenate or primary skeletal muscle cells of mice were measured using the Iron Assay Kit (Sigma, USA) and MDA assay kit (Jianchen, China), respectively. Specific operations were carried out according to the manufacturer’s instructions.

### Mitochondrial function assay

The levels of GSH and Glutathione oxidized (GSSG) in the skeletal muscle homogenate or primary skeletal muscle cells of mice were measured using the Total glutathione / Oxidized glutathione assay kit (Jianchen, China) to calculate their ratio. Additionally, the ATP content in the skeletal muscle homogenate or primary skeletal muscle cells was detected using the ATP assay kit (Jianchen, China). The redox status of cells was detected using BODIPY 581/591 C11 (MCE, USA). Specific operations were performed according to the manufacturer’s instructions.

### HE staining

Isolated skeletal muscle tissue is fixed in 4% paraformaldehyde at room temperature for 24 h, then embedded in paraffin and cut into 4-micrometer sections. After drying, the sections are immersed in xylene to dewax for 5 min, followed by rehydration, staining with hematoxylin for 10 min, washing, and differentiation with 1% hydrochloric alcohol. Subsequently, the sections are stained with eosin for 1 min, dehydrated with graded alcohol, and immersed in xylene twice for 5 min each time to make the sections transparent. Finally, the sections are mounted with neutral balsam and observed and analyzed under an optical microscope.

### Sirius red staining

To examine muscle fibers using Sirius Red staining, follow the manufacturer’s instructions. Drop-stain the pre-processed paraffin sections in Sirius Red staining solution for 30 min. Perform two rapid rinses with 0.5% dilute acetic acid. Finally, dehydrate the sections with graded alcohol, clear them three times in xylene, and mount them with neutral resin mounting medium. Observe and analyze the sections under an optical microscope.

### Immunohistochemistry (IHC)

For IHC staining, pre-processed paraffin sections are incubated with primary antibodies against Piezo1 (1:100, Affinity, China), GPX4 (1:100, Affinity, China), and superoxide dismutase (SOD) (1:100, Affinity, China). After completion, they are incubated with the corresponding secondary antibodies for 1 h, developed with DAB reagent, and finally counterstained with hematoxylin to stain the cell nuclei. Observation is performed using an optical microscope.

### Cell culture and intervention

Ten 4-week-old C57BL/6 mice were euthanized using an overdose of sodium pentobarbital injected intraperitoneally. Under sterile conditions, the skin of the hindlimb thigh was incisioned, and the thigh muscle belly was excised and placed in PBS. It was washed three times, cut into 1 mm3 pieces using sterile scissors, and placed in a digestion solution containing 0.1% Type I collagenase (Solarbio, China), 0.2% Type IV collagenase (Solarbio, China), and 0.2% Dispase II (Solarbio, China). Digestion was performed on a 37℃ shaker for 30 min, followed by centrifugation. The supernatant was discarded, and the pellet was resuspended in a six-well plate. Cells were cultured in a 37℃ incubator with 5% CO^2^. Supernatant was collected and cultured in another well every hour, and this process was repeated three times. The medium was changed after 48 h, and cells were passaged when they reached 80% confluence. Cell experiments were performed on the third passage. Primary skeletal muscle cells were placed in a Flexcell FX5000 Tension system (Flexcell International, USA) for mechanical stretch stress loading experiments. The parameters were set to a frequency of 1.0 Hz, a stretch intensity of 10%, and a duration of 12 h [30]. Cells were simultaneously treated or untreated with different concentrations of Fer-1 (MCE, USA), Mitoquinone mesylate (MCE, USA), and Gallic acid (MCE, USA) for appropriate durations.

### Cell viability assay

The cell viability of primary skeletal muscle cells after treatment with GA was determined using the CCK-8 kit (APExBIO, USA). Following the manufacturer’s instructions, primary skeletal muscle cells were treated with different concentrations of GA for 24 h and then washed three times with PBS. Subsequently, a mixture of 90 µL of culture medium and 10 µL of CCK-8 reagent was added to each well, and the cells were incubated for another 2 h. The OD value at 450 nm was measured, and the relative cell survival rate (%) was calculated.

### Transmission electron microscopy (TEM)

Cellular mitochondria were observed using transmission electron microscopy. Skeletal muscle cells were seeded in a 6-well plate and, after the intervention was completed, were preserved in electron microscopy fixative. TEM was then used to directly observe morphological changes in mitochondria among different groups.

### Immunofluorescence

After the intervention, skeletal muscle cells were fixed using 4% paraformaldehyde. They were then permeabilized with Enhanced Immunostaining Permeabilization Buffer (Beyotime, China) for 10 min at room temperature and blocked with Immunol Staining Blocking Buffer (Beyotime, China) for 90 min. Following this, the cells were incubated overnight at 4℃ with primary antibodies against Piezo1 (1:300, DF12083, Affinity, China), GPX4 (1:300, DF6701, Affinity, China), and SOD (1:300, BF8509, Affinity, China). After incubation with the primary antibodies, the cells were incubated with secondary antibodies for 2 h at room temperature in the dark. Finally, the nuclei were stained with Antifade Mounting Medium containing DAPI for 10 min. The cells were then observed using an inverted fluorescence microscope (Nikon, Japan), and the fluorescence intensity was measured using Image J software 1.53.

### ROS detection

ROS were detected using DHE (MCE, USA). Skeletal muscle cells were seeded in a 6-well plate and, after the intervention was completed, incubated in serum-free medium containing 5 µM DHE for 30 min. The medium was then aspirated, and the cells were washed three times with PBS. Finally, the cells were observed using an inverted fluorescence microscope (Nikon, Japan).

### Mitochondrial membrane potential detection (ΔΨm)

Mitochondrial membrane potential (ΔΨm) was detected using the JC-1 staining kit (Beyotime, China). According to the manufacturer’s instructions, the intervened skeletal muscle cells were incubated with the JC-1 working solution for 20 min. Following this, the cells were washed twice with JC-1 staining buffer and observed using the flow cytometer (BD, USA). Images were captured and processed for analysis.

### RNA extraction and real time quantitative PCR (RT-qPCR)

Total RNA was extracted from tissues or cells using TRIzol reagent (Vazyme, China) according to the manufacturer’s instructions. The obtained total RNA was then reverse-transcribed into cDNA using HiScript III All-in-one RT SuperMix Perfect for qPCR (Vazyme, China). Real-time quantitative PCR was performed using ChamQ SYBR qPCR Master Mix (Vazyme, China) with the following reaction mixture: 4 µl of 2×ChamQ SYBR qPCR Master Mix, 0.4 µl of each primer, and 9.2 µl of Diluted cDNA. The amplification conditions were as follows: 30 s at 95 °C followed by 40 cycles of 10 s at 95 °C and 30 s at 60 °C. The relative gene expression was determined using the 2^−△△Ct^ method and normalized to the β-actin level. Primer sequences are detailed in Table 1.

**Table 1:** Primer design sequence

Target gene	Sequence(5’-3’)
β-actin	F: GATCAGCAAGCAGGAGTACGA
	R: GGGTGTAAAACGCAGCTCA
Piezo1	F: CTTCGGGTTGGAGAGGTACG
	R: ACTCAAAGGCTCTTCGGCTC
GPX4	F: GCCTCGCAATGAGGCAAAAC
	R: CAAACTGGTTGCAGGGGAAG
COX2	F: CAGGACTCTGCTCACGAAGG
	R: ATCCAGTCCGGGTACAGTCA
FTH1	F: AAGTGCGCCAGAACTACCAC
	R: AGCCACATCATCTCGGTCAA
TOM20	F: GTGCGGTGTGTTGTCTGTTG
	R: GATGGAACACCCCAGAGACG
SOD	F: TTCTCGTCTTGCTCTCTCTGG
	R: CTTCTGCTCGAAGTGGATGGT

### Western blotting

After extracting proteins from mouse tissues or cells using RIPA lysis buffer containing 1 mM phenylmethanesulfonyl fluoride (PMSF), protein concentrations were measured using the BCA Protein Concentration Assay Kit (Beyotime, China). Proteins from each group were separated by sodium dodecyl sulfate polyacrylamide gel electrophoresis (SDS-PAGE) and then transferred to 0.45 μm polyvinylidene fluoride membranes (Millipore, USA). The membranes were blocked with QuickBlock™ Western Buffer (Beyotime, China) for 1 h and then incubated with primary antibodies against β-actin (1:5000, AF7018, Affinity, China), Piezo1 (1:1000, ab128245, Abacm, USA), GPX4 (1:1000, DF6701, Affinity, China), FTH1 (1:1000, DF6278, Affinity, China), COX2 (1:1000, AF7003, Affinity, China), TOM20 (1:1000, AF5206, Affinity, China), and SOD (1:2000, AF5198, Affinity, China) overnight at 4℃. Subsequently, the blots were incubated with the corresponding secondary antibodies. Finally, the blots were detected using Enhanced chemiluminescence (ECL) detection reagents (Vazyme, China). The gray values of the protein bands were quantitatively analyzed using Image J software 1.53.

### Statistical analysis

All experiments were performed with at least three biological replicates. All statistical analyses were conducted using Graphpad Prim 9.5. Data are presented as mean ± standard deviation (SD). All data met the assumption of normal distribution. To compare between two groups, t-tests (two-tailed) were used. For comparisons among three or more groups, one-way analysis of variance (ANOVA) with Tukey’s test was employed. Relative protein expression from Western blot analysis was quantified using ImageJ software, normalizing to β-actin levels for each sample. Immunohistochemical analysis was performed by quantifying the integrated optical density (IOD) per area using ImageJ software to evaluate SOD and other marker expression levels. Quantify the average fluorescence intensity using ImageJ software, and calculate the average fluorescence intensity of each indicator in the immunofluorescence experiment by dividing the total fluorescence intensity of a specific region by its corresponding area. A value of *P* < 0.05 was considered statistically significant. Experimental results were processed using Adobe Illustrator 2022 and Adobe Photoshop cc 2019.

## Results

### GA alleviates skeletal muscle injury and ferroptosis caused by excessive exercise in mice

**Fig. 1:**
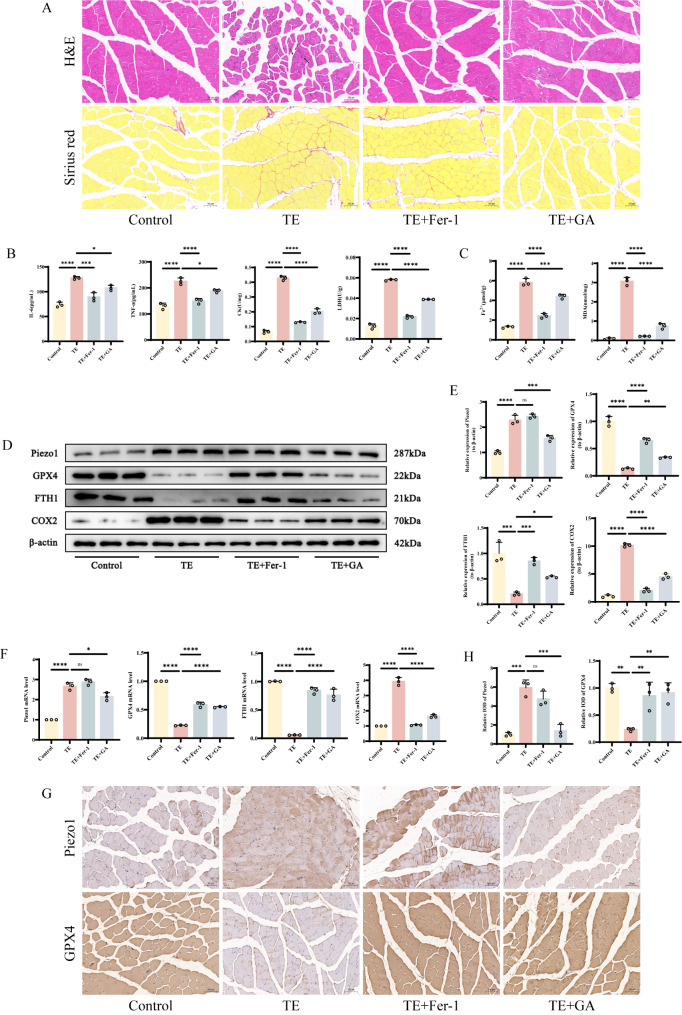
GA alleviates skeletal muscle injury and ferroptosis caused by excessive exercise in mice **A** Images of mouse skeletal muscle stained with H&E and Sirius red, “_” represents 100 μm **B-C** Detection of IL-6, TNF-α, CK, LDH, Fe^2+^ and MDA levels in skeletal muscle tissue by ELISA **D-E** Western blot bands represent the expression levels of Piezo1, GPX4, FTH1 and COX2 proteins in different groups of mouse tissues, with histograms representing the quantitative data of each index standardized to β-actin **F** Detection of Piezo1, GPX4, FTH1 and COX2 mRNA levels in skeletal muscle tissue by RT-qPCR **G-H** Immunohistochemical detection of Piezo1 and GPX4 expression in skeletal muscle tissue of different groups of mice, with “_” indicating 100 μm, accompanied by quantitative histograms Values are expressed as the mean ± SD from three independent experiments (*n* = 3). *p < 0.05, **p < 0.01, ***p < 0.001, **** *p* < 0.0001

We investigated the effect of GA on skeletal muscle injury caused by excessive exercise and its possible mechanism in vivo. We used a treadmill to establish an exercise injury model in mice. Hematoxylin and eosin (HE) and Sirius red staining revealed that after treadmill exercise, the muscle fibers in the TE group were disorganized compared to the Control group, with edema in a large number of muscle cells, obvious inflammatory cell infiltration, and partial rupture of the fascial membrane. However, after intervention with the ferroptosis inhibitor Fer-1 or GA, the muscle fibers were more orderly arranged, and some muscle cells exhibited mild edema and a small amount of inflammatory cell infiltration (Fig. 1A). Furthermore, the levels of inflammatory damage markers, including IL-6, CK, LDH, and TNF-α, were significantly elevated in the skeletal muscle homogenate of mice in the TE group compared to the Control group, but treatment with Fer-1 and GA significantly reduced the levels of these inflammatory markers, which may be attributed to their inhibitory effects on ferroptosis (Fig. 1B). Based on these findings, we observed a sharp increase in the expression of Fe^2+^ and MDA in the skeletal muscle of mice after TE intervention, while GA was able to reduce their expression, similar to the effect of Fer-1 intervention (Fig. 1C). Western blot analysis showed that compared to the Control group, the expression of Piezo1 (a mechanically sensitive cation channel) and cyclooxygenase-2 (COX2) was increased, while the expression of GPX4 and Ferritin Heavy Chain 1 (FTH1) was decreased in the skeletal muscle tissue of mice in the TE group. There was no significant difference in Piezo1 expression between the TE + Fer-1 group and the TE group, while COX2 was decreased and the expression of GPX4 and FTH1 was increased. After GA intervention, both Piezo1 and COX2 were significantly decreased, and the expression of GPX4 and FTH1 was increased (Fig. 1D-E). Similar trends were observed in the RT-qPCR results (Fig. 1F). Immunohistochemical detection of Piezo1 and GPX4 positive cell expression also showed similar trends (Fig. 1G-H). These findings clearly indicate that GA can alleviate skeletal muscle injury induced by excessive exercise by inhibiting ferroptosis.

### GA attenuates oxidative stress injury in mice with EIMD

**Fig. 2:**
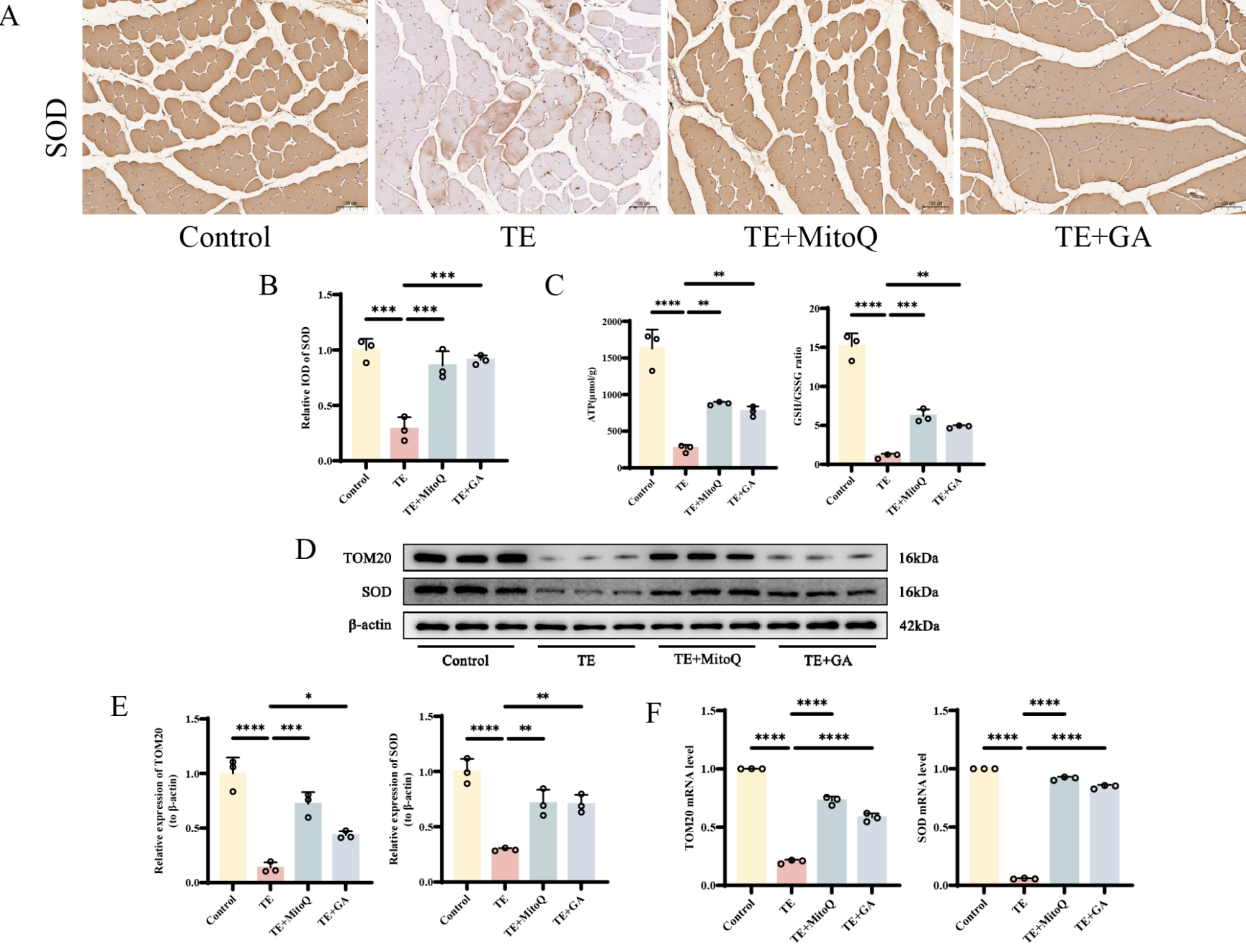
GA alleviates mitochondrial oxidative stress induced by excessive exercise in mice **A-B** Immunohistochemical analysis of SOD expression in skeletal muscle tissue from different groups of mice, with “_” indicating 100 μm, accompanied by quantitative histograms **C** Detection of ATP, GSH and GSSG levels in skeletal muscle tissue by ELISA **D-E** Western blot bands represent the expression levels of TOM20 and SOD proteins in different groups of mouse tissues, with histograms representing the quantitative data of each index standardized to β-actin **F** Detection of TOM20 and SOD mRNA levels in skeletal muscle tissue by RT-qPCR Values are expressed as the mean ± SD from three independent experiments (*n* = 3). * *p* < 0.05, **p < 0.01, *** *p* < 0.001, **** *p* < 0.0001

The strong correlation between mitochondrial oxidative stress and ferroptosis has been fully demonstrated. Therefore, after confirming the existence of ferroptosis, we further explored the mechanism of mitochondrial oxidative stress in skeletal muscle injury caused by excessive exercise and the intervention effect of GA. Immunohistochemical detection of SOD-positive cells in mouse skeletal muscle showed that the ratio of SOD-positive cells in the TE group was significantly lower than that in the control group. However, after intervention with mitochondrial-targeted antioxidant Mitoquinone mesylate and GA, the SOD content was significantly increased (Fig. 2A-B). In addition, ATP production in skeletal muscle was significantly reduced after excessive exercise intervention, while MitoQ and GA could partially increase ATP production (Fig. 2C). Simultaneously, we also found changes in redox homeostasis in skeletal muscle tissue. Compared with the Control group, the expression of GSH in skeletal muscle in the TE group was extremely low, and the GSH/GSSG ratio was significantly reduced (Fig. 2D). Western blot and RT-qPCR were used to detect the protein and mRNA expression levels of mitochondrial membrane proteins TOM20 and SOD. The expression of TOM20 and SOD in the TE group was significantly lower than that in the Control group, while their expression in the TE + MitoQ and TE + GA groups was significantly higher than that in the TE group (Fig. 2E-F). These findings suggest that GA can alleviate skeletal muscle injury induced by excessive exercise by inhibiting mitochondrial oxidative stress.

### Mechanical stress overload mediates skeletal muscle cell Injury and Ferroptosis

**Fig. 3:**
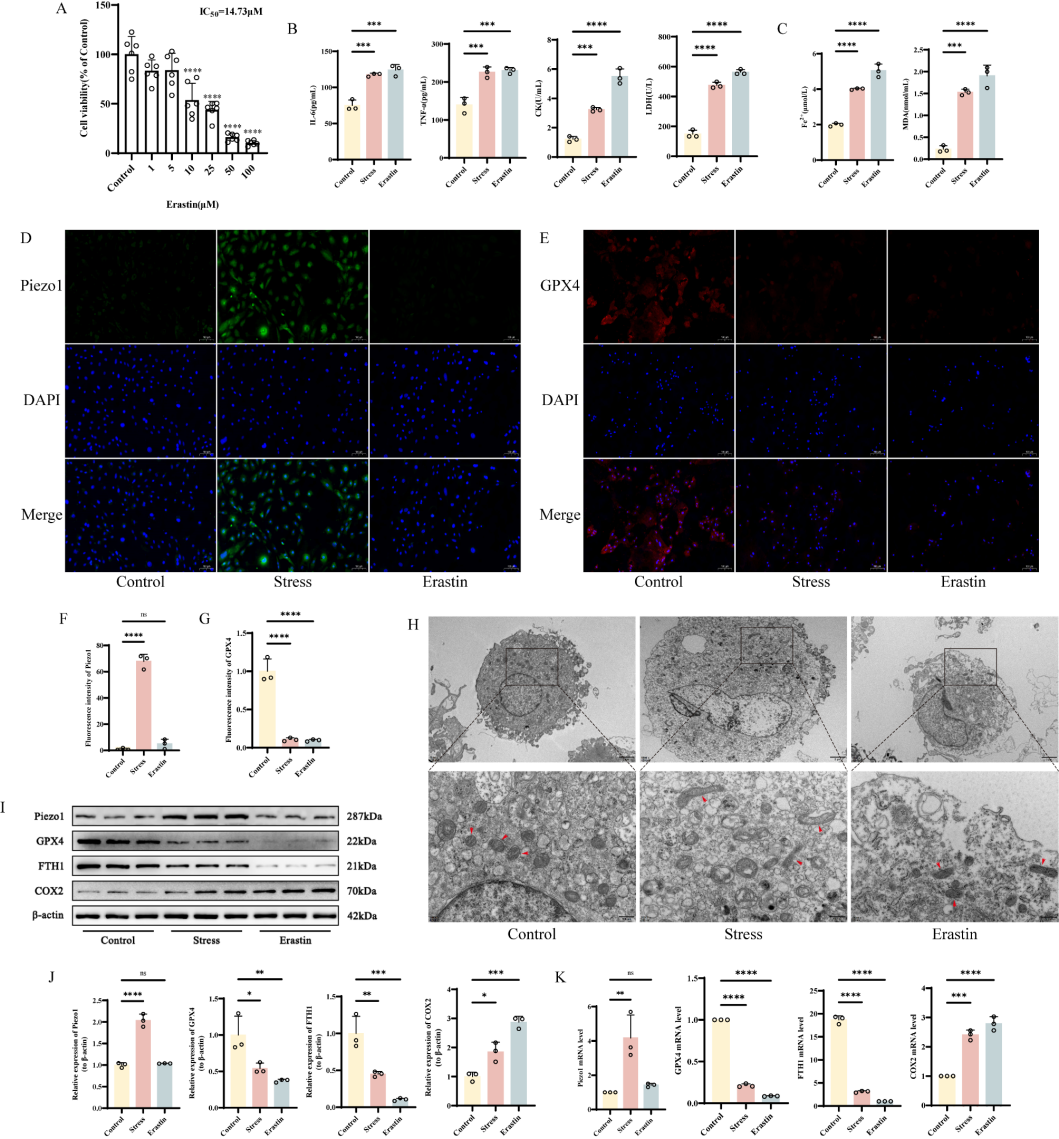
Mechanical stress overload mediates skeletal muscle cell injury and ferroptosis **A** CCK-8 assay histograms showing the cytotoxicity of Erastin on skeletal muscle cells **B-C** Detection of IL-6, TNF-α, CK, LDH, Fe^2+^ and MDA levels in skeletal muscle tissue by ELISA **D-G** Immunofluorescence detection of Piezo1 and GPX4 expression in different groups of cells, with “_” representing 100 μm, accompanied by quantitative histograms **H** Mitochondrial morphology in different groups of cells under transmission electron microscopy, with “_” indicating 500 nm **I-J** Western blot bands represent the expression levels of Piezo1, GPX4, FTH1 and COX2 proteins in different groups of mouse cells, with histograms representing the quantitative data of each index standardized to β-actin **K** Detection of Piezo1, GPX4, FTH1 and COX2 mRNA levels in skeletal muscle cells by RT-qPCR Values are expressed as the mean ± SD from three independent experiments (*n* = 3). * *p* < 0.05, **p < 0.01, *** *p* < 0.001, **** *p* < 0.0001

To further clarify the underlying mechanism of skeletal muscle injury induced by excessive exercise, we conducted a series of experimental validations on primary skeletal muscle cells. Firstly, we determined the ideal concentration of ferroptosis inducer Erastin for primary skeletal muscle cells by detecting cell viability using the CCK-8 assay (Fig. 3A). Based on this, we subjected skeletal muscle cells to mechanical stretch stress stimulation and subsequently measured the expression of damage markers, including IL-6, TNF-α, CK, and LDH, in the supernatants of each group. The results showed that the expression of these markers was significantly elevated compared to the Control group (Fig. 3B-C). Additionally, ferroptosis-related markers such as Fe^2+^ and MDA levels also exhibited a similar trend in both groups (Fig. 3C). Immunofluorescence results suggested that the fluorescence intensity of GPX4 was significantly reduced in both the stress group and the Erastin group compared to the Control group, while Piezo1 expression was only enhanced in the stress group (Fig. 3D-G). TEM observation revealed that after interventions in both groups, most mitochondria in skeletal muscle cells exhibited reduced volume, increased density, and decreased mitochondrial ridges (Fig. 3H). The protein and mRNA expression levels of GPX4 and FTH1 were significantly lower in both the stress group and the Erastin group compared to the Control group, while the expression level of COX2 increased. Piezo1 expression was only upregulated in the stress group (Fig. 3I-K). In summary, these results indicate that excessive exercise causes damage to skeletal muscle cells by inducing ferroptosis.

### GA alleviates cellular injury caused by mechanical stress overload by inhibiting ferroptosis in skeletal muscle cells

**Fig. 4:**
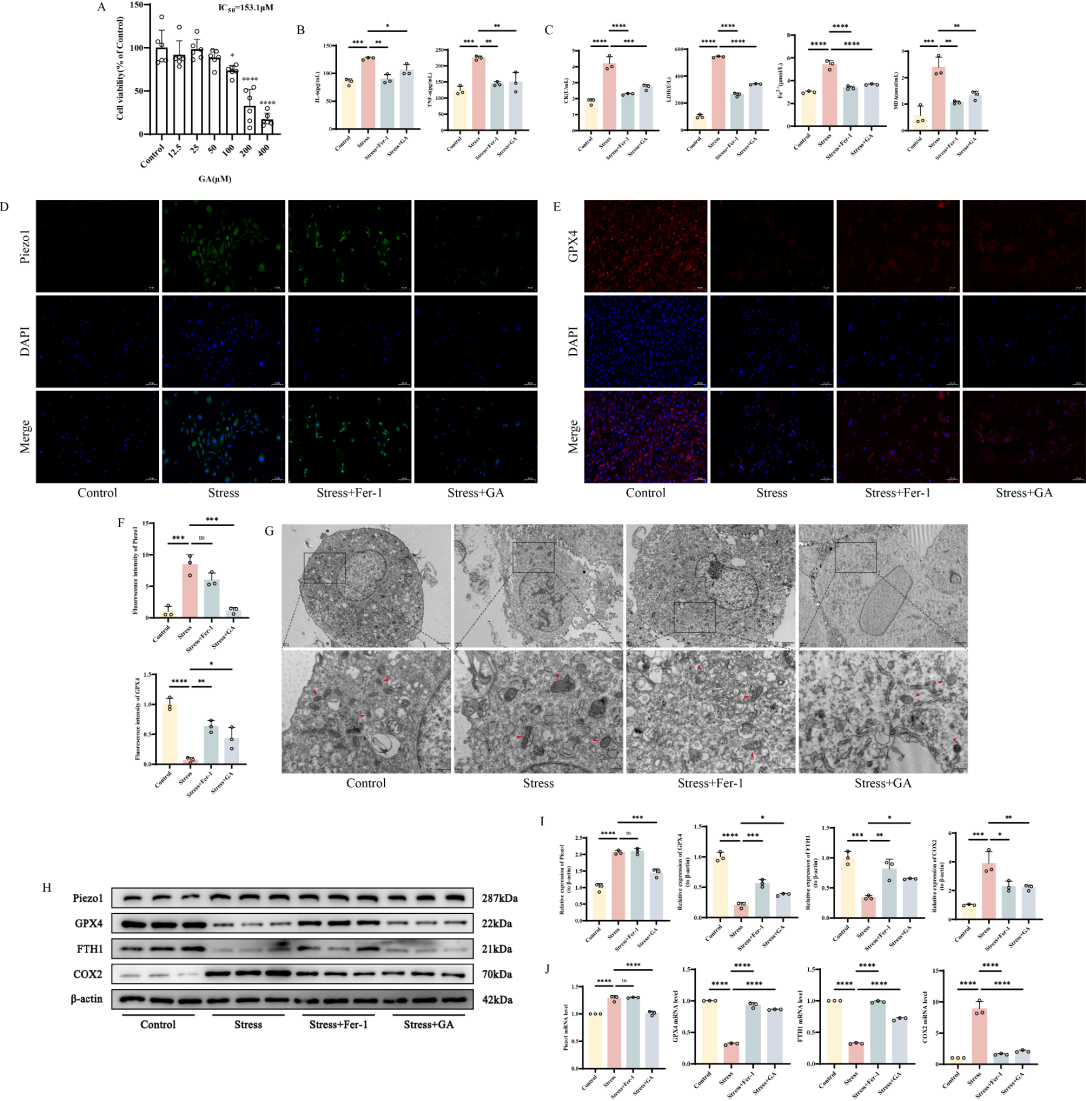
GA alleviates cellular injury caused by mechanical stress overload by inhibiting ferroptosis in skeletal muscle cells **A** CCK-8 assay histograms showing the cytotoxicity of GA on skeletal muscle cells **B-C** Detection of IL-6, TNF-α, CK, LDH, Fe^2+^ and MDA levels in skeletal muscle tissue by ELISA **D-F** Immunofluorescence detection of Piezo1 and GPX4 expression in different groups of cells, with “_” representing 100 μm, accompanied by quantitative histograms **G** Mitochondrial morphology in different groups of cells under transmission electron microscopy, with “_” indicating 500 nm **H-I** Western blot bands represent the expression levels of Piezo1, GPX4, FTH1 and COX2 proteins in different groups of mouse cells, with histograms representing the quantitative data of each index standardized to β-actin **J** Detection of Piezo1, GPX4, FTH1 and COX2 mRNA levels in skeletal muscle cells by RT-qPCR Values are expressed as the mean ± SD from three independent experiments (*n* = 3). * *p* < 0.05, **p < 0.01, *** *p* < 0.001, **** *p* < 0.0001

To clarify the potential mechanism of GA’s effect on skeletal muscle cell injury caused by mechanical stress overload and its association with ferroptosis, we conducted corresponding validations in vitro. Firstly, we determined the toxic effects of different concentrations of GA on primary skeletal muscle cells using the CCK-8 assay to establish its dosing concentration. At 50 µM, no significant cell toxicity was observed, while a significant decrease in cell viability was noted at 100 µM (Fig. 4A). Therefore, we selected 50 µM as the subsequent dosing concentration for subsequent experiments. We found that GA intervention exhibited similar effects to Fer-1, with significant decreases in damage markers such as IL-6, TNF-α, CK, and LDH, as well as ferroptosis markers such as Fe^2+^ and MDA, in the cell supernatants (Fig. 4B-C). GPX4 fluorescence intensity was significantly increased compared to the stress group, while Piezo1 fluorescence intensity decreased only after GA intervention (Fig. 4D-F). TEM observations revealed that mitochondria in cells treated with Fer-1 or GA displayed relatively clearer outer membrane boundaries, more intact cristae structures, and a relatively decreased internal density compared to those in the stress group (Fig. 4G). The protein and mRNA expression levels of GPX4 and FTH1 in skeletal muscle cells after GA or Fer-1 intervention were significantly increased compared to the stress group, while the expression level of COX2 decreased. Piezo1 expression was only downregulated after GA intervention (Fig. 4H-J). Therefore, we hypothesize that GA is a potential therapeutic agent for EIMD, likely exerting its effects by reducing oxidative stress damage and the expression of ferroptosis-related markers induced by mechanical stress.

### Mechanical stress overload leads to ROS Accumulation and mitochondrial oxidative stress in skeletal muscle cells

**Fig. 5:**
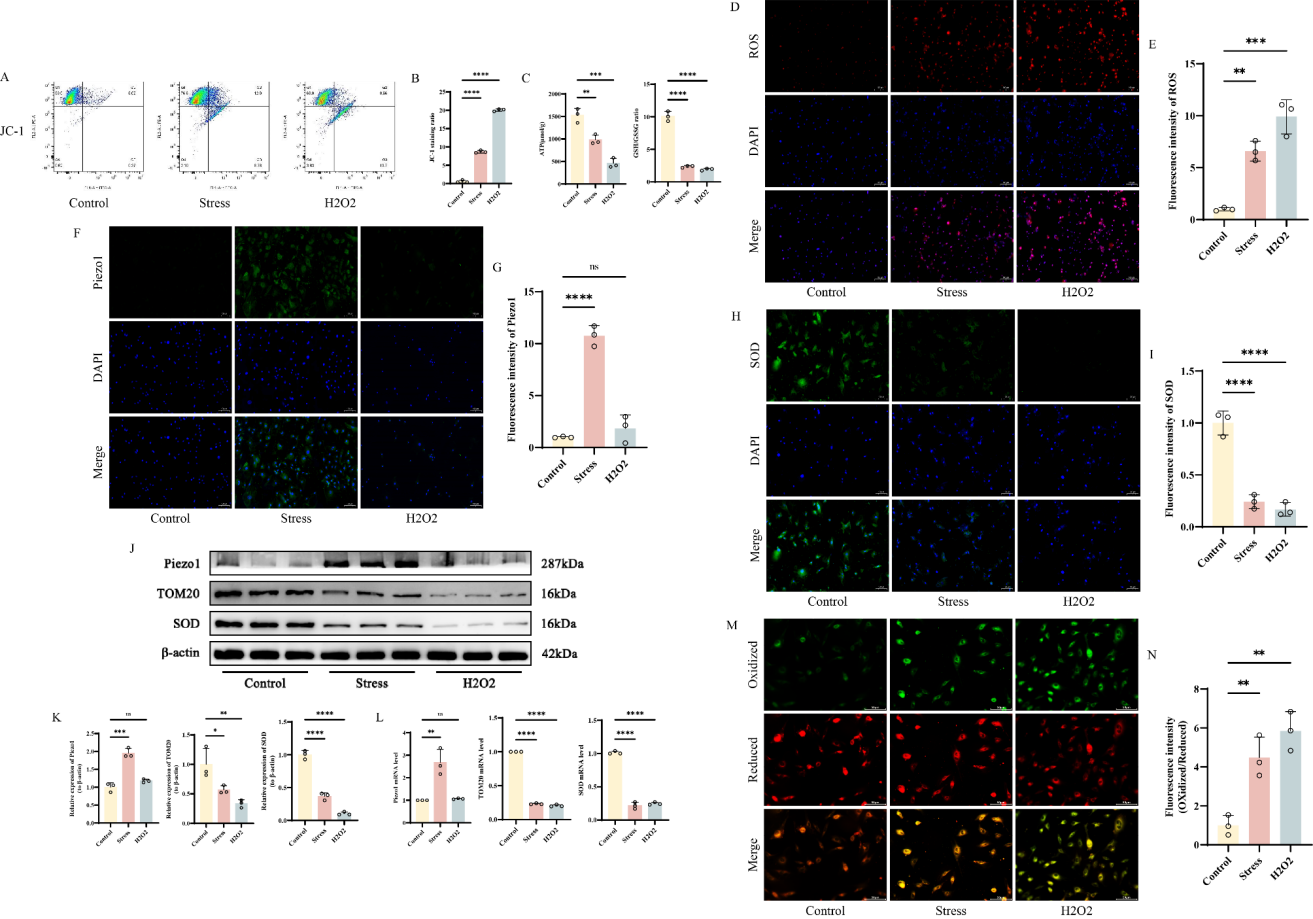
Mechanical stress overload leads to ros accumulation and mitochondrial oxidative stress in skeletal muscle cells **A-B** Flow cytometry plots and analysis bar graphs of different groups of cells detected by JC-1 **C** Detection of ATP, GSH and GSSG levels in skeletal muscle tissue by ELISA**D-E** DHE detection of ROS fluorescence images and analysis bar graphs in different groups of cells, with “_” representing 100 μm **F-I** Immunofluorescence detection of Piezo1 and SOD expression in different groups of cells, with “_” representing 100 μm, accompanied by quantitative histograms **J-K** Western blot bands represent the expression levels of Piezo1, TOM20 and SOD proteins in different groups of mouse cells, with histograms representing the quantitative data of each index standardized to β-actin **L** Detection of Piezo1, TOM20 and SOD mRNA levels in skeletal muscle cells by RT-qPCR**M-N** Images of mouse skeletal muscle cells stained with C11-BODIPY 581/591 probe, with “_” representing 100 μm, accompanied by corresponding histograms of fluorescence intensity ratio (oxidized/reduced) Values are expressed as the mean ± SD from three independent experiments (*n* = 3). * *p* < 0.05, **p < 0.01, *** *p* < 0.001, **** *p* < 0.0001

Detection of ΔΨm in skeletal muscle cells using the JC-1 fluorescent probe revealed a significant increase in the green fluorescence ratio after stress or H2O2 intervention compared to the Control group, indicating that both interventions disrupt the ΔΨm level in skeletal muscle cells (Fig. 5A, B). Additionally, detection of ROS in cells using DHE showed a significant increase in ROS levels in stressed and H2O2-treated cells compared to the control group (Fig. 5D-E). Following intervention, ATP production and the GSH/GSSG ratio in cells also decreased significantly (Fig. 5C). SOD fluorescence intensity was significantly lower compared to the control group, while Piezo1 fluorescence intensity only increased after stress intervention (Fig. 5F-I). Western blot and RT-qPCR were used to detect the protein and mRNA expression levels of TOM20 and SOD. Compared to the Control group, the expression of TOM20 and SOD was significantly reduced in both the stress and H2O2 groups, while Piezo1 expression only increased in the stress group (Fig. 5J-L). Finally, the redox status of primary skeletal muscle cells was detected using the BODIPY 581/591 C11 probe. The results showed that both stressed and H2O2-treated skeletal muscle cells exhibited a shift from red to green fluorescence, indicating a significant increase in oxidation levels in these cells (Fig. 5M-N). By comparing various indicators with the H2O2-induced model group, mechanical stress stimulation was found to enhance the expression of oxidative stress-related markers in skeletal muscle cells and cause mitochondrial damage, which may be one of the underlying mechanisms contributing to skeletal muscle injury.

### GA can alleviate mitochondrial oxidative stress injury in skeletal muscle cells caused by mechanical stress overload

**Fig. 6:**
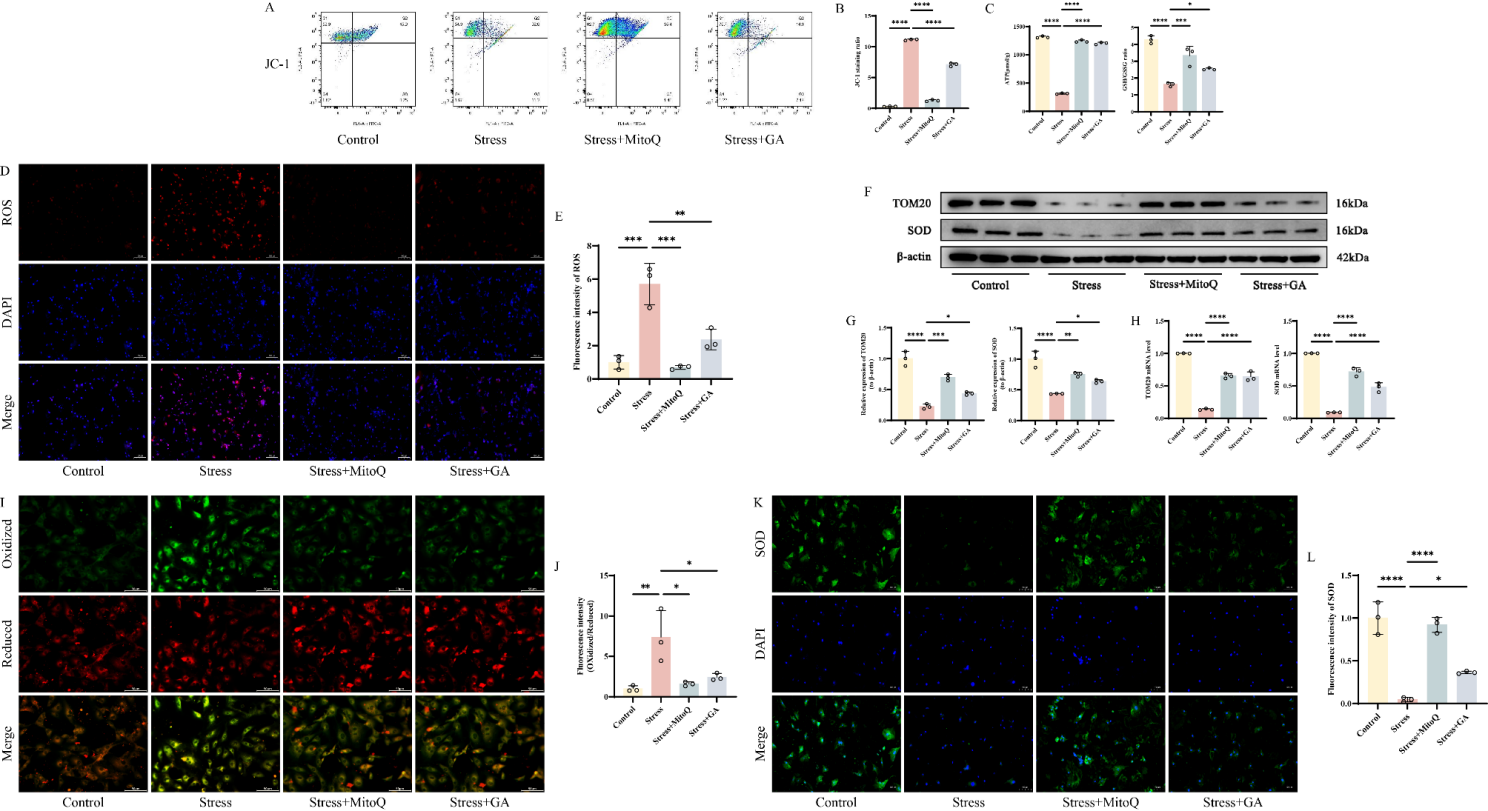
GA can alleviate mitochondrial oxidative stress injury in skeletal muscle cells caused by mechanical stress overload **A-B** Flow cytometry plots and analysis bar graphs of different groups of cells detected by JC-1 **C** Detection of ATP, GSH and GSSG levels in skeletal muscle tissue by ELISA **D-E** DHE detection of ROS fluorescence images and analysis bar graphs in different groups of cells, with “_” representing 100 μm **F-G** Western blot bands represent the expression levels of TOM20 and SOD proteins in different groups of mouse cells, with histograms representing the quantitative data of each index standardized to β-actin**H** Detection of TOM20 and SOD mRNA levels in skeletal muscle cells by RT-qPCR **I-J** Images of mouse skeletal muscle cells stained with C11-BODIPY 581/591 probe, with “_” representing 100 μm, accompanied by corresponding histograms of fluorescence intensity ratio (oxidized/reduced) **K-L** Immunofluorescence detection of SOD expression in different groups of cells, with “_” representing 100 μm, accompanied by quantitative histograms Values are expressed as the mean ± SD from three independent experiments (*n* = 3). * *p* < 0.05, **p < 0.01, *** *p* < 0.001, **** *p* < 0.0001

Finally, we validated the protective effect of GA on mitochondrial oxidative stress injury in skeletal muscle cells caused by mechanical stress overload through in vitro experiments. We found that GA intervention had a similar protective effect on mitochondria as MitoQ. Specifically, GA partially reversed the disruption of ΔΨm and the decrease in ATP production and GSH/GSSG ratio in skeletal muscle cells under mechanical stress overload (Figs. 6A-C). GA treatment also reduced the accumulation of ROS in stressed cells, with a significant decrease in DHE staining intensity (Fig. 6D-E). Western blot and RT-qPCR analysis of TOM20 and SOD protein and mRNA expression levels confirmed that GA intervention significantly increased the expression of TOM20 and SOD (Fig. 6F-H). BODIPY 581/591 C11 detection revealed that the fluorescence changed partially from green to red after GA intervention (Fig. 6I-J), indicating stronger reducing properties of skeletal muscle cells under GA treatment. Immunofluorescence showed that compared to the stress group, the fluorescence intensity of SOD significantly increased after GA or MitoQ intervention (Fig. 6K-L). These findings suggest that GA exerts its antioxidant effects and provides protective benefits to the mitochondria in skeletal muscle cells.。.

**Fig. 7:**
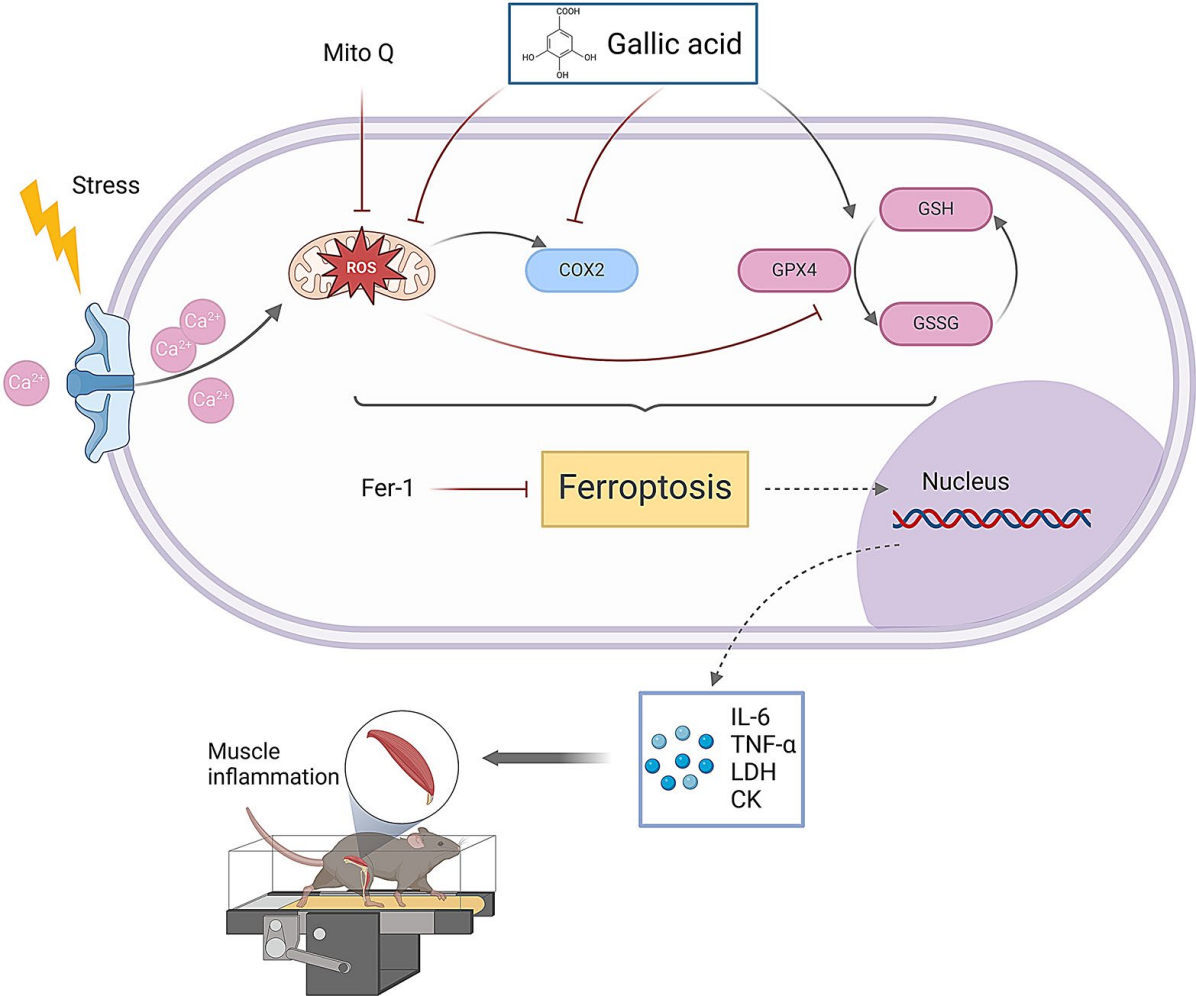
Mechanism schematic diagram of this study Gallic acid inhibits mechanical stress overload-induced skeletal muscle injury via ferroptosis-related pathways by exerting its antioxidant function

## Discussion

GA is a phenolic extract derived from natural plant sources with a wide range of anti-inflammatory and antioxidant activities [31]. Our study reveals the ability of GA to protect skeletal muscle from inflammation and injury induced by strenuous exercise in vivo and in vitro. Specifically, GA attenuated EIMD in the lower limbs of C57 mice and abrogated excessive exercise-induced mitochondrial ROS escape, oxidative stress, and ferroptosis in skeletal muscle cells. GA was shown in our study to have good antioxidant properties in skeletal muscle cells, which can counteract the mitochondrial oxidative stress induced by strenuous exercise and maintain mitochondrial functional and structural stability, as well as act as a ROS-mediated ferroptosis antagonist in cells.

The benefits and drawbacks of exercise-induced ROS production on skeletal muscle health may depend on the balance between the level of ROS produced during exercise and the ability of the cellular antioxidant system to protect the cell from oxidative stress damage. EIMD-related aseptic injury leads to early ROS production by mitochondria, and these organelles undergo rapid subsequent destruction, with concomitant damage to the ultrastructure of the muscle and the corresponding release of intracellular damage-associated molecular patterns (DAMP) being released accordingly, leading to swelling of the affected limb, impaired muscle regeneration, and reduced exercise capacity [32]. Notably, GA could reduce myocyte oedema and inflammatory infiltration, improve myofibre rupture caused by overexercise and normalize the structural morphology of skeletal muscle, which initially proved the good role of GA in skeletal muscle repair. Studies have shown that myofibrillar disarray accompanied by loss of Z-lines, abnormal mitochondrial morphology, and myofilm disruption are the hallmarks of skeletal muscle injury, while myostatin, including CK and LDH, is released into the bloodstream [33]. CK is a damage-associated factor released due to muscle fiber breakdown, often accompanied by symptoms of muscle tightness and pain. Clinical studies have shown that eccentric contraction exercises in skeletal muscle can lead to elevated CK levels [34]. Meanwhile, CK is also regarded as a qualitative marker of skeletal muscle microtrauma, with its expression levels influenced by factors such as exercise duration and training intensity [35]. Additionally, LDH serves as an indicator of various stimuli affecting skeletal muscle, including physical exercise, weight-bearing activities, and estrogen levels. It catalyzes the interconversion of pyruvate and lactate to meet cellular energy demands, and its activity is closely linked to muscle fatigue [36]. Therefore, we used ELISA experiments to detect inflammatory factors and injury markers in the blood. The results indicated that GA exhibited similar effects to Fer-1 in alleviating inflammation and muscle damage in mice. Fer-1 is a specific inhibitor of ferroptosis. Wang and others have demonstrated that ferroptosis is a form of cell death that plays an important role in pathological changes such as skeletal muscle inflammation, injury, and atrophy. Based on our experimental results, it can be suggested that ferroptosis may be one of the targets of GA in inhibiting EIMD in mice [37].

The activation of ferroptosis hinders the regeneration of skeletal muscle in mice. Previous studies have found that an imbalance in iron homeostasis can lead to diseases such as skeletal muscle atrophy and age-related muscle loss in mice [38]. Notably, GPX4 is a key marker of ferroptosis and acts as a terminal terminator that catalyses the transformation of harmful lipid peroxides into pentatoxic lipid alcohols [39]. We measured the important products of lipid peroxidation and iron imbalance, i.e., MDA and Fe^2+^, in tension-stimulated skeletal muscle cells and showed that GA significantly attenuated the inflammatory response induced by tension loading, reduced the MDA and Fe^2+^ content, and exhibited the same effect as Fer-1. In addition, GA increased the low expression of GPX4 and FTH1 in skeletal muscle cells under pathological conditions. FTH1 is an inhibitor of the cytoplasmic iron storage protein complex, which can play an important antioxidant role in cells by chelating redox-activated iron and regulating iron homeostasis in various types of cells [40]. The above results can demonstrate that ferroptosis is one of the important targets of GA for the treatment of EIMD in mice.

Oxidative stress is strongly correlated with ferroptosis, and oxidative stress and cellular antioxidant levels are important regulators of lipid peroxidation in ferroptosis, which was verified in our experiments by changes in GSH/GSSG ratios in EIMD intervened by the antioxidant MitoQ [41]. Similarly, given the important role of mitochondria in the oxidative stress response, we investigated mitochondrial morphology and function in mouse skeletal muscle. Transmission electron microscopy results showed that mitochondria in skeletal muscle cells intervened by mechanical force pulling exhibited volume collapse, increased density, and reduced mitochondrial ridges, implying that there is a deleterious effect of stress loading on cellular mitochondria, which could be ameliorated by Fer-1, an inhibitor of ferroptosis, in combination with GA to improve this situation. On this basis, we found that GA significantly increased the expression of mitochondrial membrane proteins TOM20 and SOD in muscle tissues of EIMD mice and in traction-injured skeletal muscle cells, and the results were similar to those of the MitoQ intervention, which demonstrated the powerful antioxidant capacity of GA [42]. When referring to mitochondrial function and oxidative stress response, we detected changes in ATP and ROS levels in skeletal muscle cells, and as expected, GA promoted ATP production and reduced ROS content in damaged muscle cells, demonstrating its maintenance of mitochondrial function and protection against oxidative stress damage, and the results of the BODIPY 581/591 C11 probe assay could also prove this point.

In our previous studies on knee cartilage and synovium, it was concluded that Piezo1, a mechanosensitive channel protein present on the cell surface, can convert the perceived physical stress into a bioelectrical signal, which allows a large amount of Ca^2+^ to enter the cell, leading to an imbalance in calcium homeostasis [43]. Subsequent stimulation of prolonged and irreversible opening of the mitochondrial permeability transition pore leads to excessive escape of ROS [44]. Therefore, we constructed stress overload mice and skeletal muscle cell models in the present study, also by running table training and mechanical distraction, and in agreement with our previous study, Piezo1 protein expression in skeletal muscle also changed in response to stress stimulation and could be intervened by GA. We therefore hypothesised that the Piezo1 protein may be one of the intermediate mechanisms by which excessive exercise induces mitochondrial ROS production. Following the activation of Piezo1 expression under excessive mechanical stress, the influx of Ca²⁺ into cells disrupts mitochondrial function, alters the expression of oxidative stress-related factors such as SOD, and triggers ferroptosis in chondrocytes through increased intracellular ROS levels and the suppression of GPX4 and FTH1 expression. These changes were mitigated by Fer-1 and GA treatment. Moreover, the downregulation of inflammatory factors, including COX2, IL-6, and TNF-α, further demonstrated the protective effects of these compounds on skeletal muscle.

Our study has some limitations, firstly, we only discussed the oxidative stress injury and ferroptosis activation caused by excessive exercise, but we did not investigate the iron disorders in skeletal muscle cells. Secondly, this research only examined the therapeutic effects of GA on oxidative stress injury and ferroptosis in EIMD, lacking detailed mechanistic pathway investigations. Although studies on GA in skeletal muscle-related diseases are limited, its antioxidant properties have been widely recognized. For instance, in studies on acute hepatic encephalopathy in rats, GA has been shown to protect liver and brain tissues by reducing oxidative stress and apoptosis through modulation of the NF-κB signaling pathway [45]. Another study on gouty arthritis in mice demonstrated that GA intervention can block NOD-like receptor thermal protein domain associated protein 3 inflammasome activation, promote nuclear factor erythroid 2-related factor 2 expression, and reduce ROS production, thereby alleviating joint swelling and synovial inflammation [20]. These findings provide a solid research foundation for GA’s antioxidative effects and, combined with our results, suggest its significant translational potential in skeletal muscle-related diseases. In addition, due to the widely proven anti-inflammatory effects of GA, our study only indicates that the ferroptosis and oxidative stress response of skeletal muscle cells caused by vigorous exercise can be effectively alleviated by GA. But the recovery of skeletal muscle inflammation injury may be indirectly affected by its systemic anti-inflammatory effect. Lastly, while our study demonstrates that GA significantly reduces inflammatory markers in EIMD, the lack of a GA-alone intervention group makes it challenging to fully distinguish its ferroptosis-inhibitory effects from its non-specific anti-inflammatory properties. In conclusion, future experiments should aim to explore the mechanisms underlying ROS generation and iron metabolism disorders, as well as the specific pathways through which GA intervenes in EIMD. These studies could provide additional insights into potential therapeutic strategies for EIMD.

## Conclusion

The findings of this experimental study indicate that GA can effectively protect the morphology and function of mitochondria in skeletal muscle cells, reduce the production of mitochondrial ROS, and subsequently alleviate mitochondrial oxidative stress. We also observed that GA intervention inhibited ferroptosis in skeletal muscle and reduced the expression of inflammatory factors and damage markers, which may be attributed to GA’s antioxidant capacity to decrease ROS levels. This leads to the protection of skeletal muscle injury caused by excessive exercise. (Fig. 7) Therefore, we believe that GA may be used in the prevention and treatment of sports-related injuries in the future.

## Electronic supplementary material

Below is the link to the electronic supplementary material.

Supplementary Material 1

Supplementary Material 2

## Data Availability

The original data and methods of all studies included in the article can be directly contacted with the corresponding author if you want to further query.

## References

[1] Froelicher VF, Duarte GM, Oakes DF, Klein J, Dubach PA, Janosi A. The prognostic value of the exercise test. Dis Mon. 1988;34:677–735. 3056676 10.1016/0011-5029(88)90011-9

[2] Fatouros IG, Jamurtas AZ. Insights into the molecular etiology of exercise-induced inflammation: opportunities for optimizing performance. J Inflamm Res. 2016;9:175–86. 27799809 10.2147/JIR.S114635 PMC5085309

[3] Schieber M, Chandel NS. ROS function in redox signaling and oxidative stress. Curr Biol. 2014;24:R453–462. 24845678 10.1016/j.cub.2014.03.034 PMC4055301

[4] Powers SK, Deminice R, Ozdemir M, Yoshihara T, Bomkamp MP, Hyatt H. Exercise-induced oxidative stress: friend or foe? J Sport Health Sci. 2020;9:415–25. 32380253 10.1016/j.jshs.2020.04.001 PMC7498668

[5] Zhou Y, Zhang X, Baker JS, Davison GW, Yan X. Redox signaling and skeletal muscle adaptation during aerobic exercise. iScience. 2024;27:109643. 38650987 10.1016/j.isci.2024.109643 PMC11033207

[6] Sciorati C, Rigamonti E, Manfredi AA, Rovere-Querini P. Cell death, clearance and immunity in the skeletal muscle. Cell Death Differ. 2016;23:927–37. 26868912 10.1038/cdd.2015.171 PMC4987728

[7] Kajarabille N, Latunde-Dada GO. Programmed cell-death by Ferroptosis: antioxidants as Mitigators. Int J Mol Sci. 2019;20:4968. 31597407 10.3390/ijms20194968 PMC6801403

[8] Zhang Y, Swanda RV, Nie L, Liu X, Wang C, Lee H, et al. mTORC1 couples cyst(e)ine availability with GPX4 protein synthesis and ferroptosis regulation. Nat Commun. 2021;12:1589. 33707434 10.1038/s41467-021-21841-w PMC7952727

[9] Agrawal S, Fox J, Thyagarajan B, Fox JH. Brain mitochondrial iron accumulates in Huntington’s disease, mediates mitochondrial dysfunction, and can be removed pharmacologically. Free Radic Biol Med. 2018;120:317–29. 29625173 10.1016/j.freeradbiomed.2018.04.002 PMC5940499

[10] Li D, Wang Y, Dong C, Chen T, Dong A, Ren J, et al. CST1 inhibits ferroptosis and promotes gastric cancer metastasis by regulating GPX4 protein stability via OTUB1. Oncogene. 2023;42:83–98. 36369321 10.1038/s41388-022-02537-x PMC9816059

[11] Chen G-H, Song C-C, Pantopoulos K, Wei X-L, Zheng H, Luo Z. Mitochondrial oxidative stress mediated Fe-induced ferroptosis via the NRF2-ARE pathway. Free Radic Biol Med. 2022;180:95–107. 35045311 10.1016/j.freeradbiomed.2022.01.012

[12] Li C, Zhang Y, Liu J, Kang R, Klionsky DJ, Tang D. Mitochondrial DNA stress triggers autophagy-dependent ferroptotic death. Autophagy. 2021;17:948–60. 32186434 10.1080/15548627.2020.1739447 PMC8078708

[13] Guerrero-Hue M, García-Caballero C, Palomino-Antolín A, Rubio-Navarro A, Vázquez-Carballo C, Herencia C, et al. Curcumin reduces renal damage associated with rhabdomyolysis by decreasing ferroptosis-mediated cell death. FASEB J. 2019;33:8961–75. 31034781 10.1096/fj.201900077R

[14] Dächert J, Ehrenfeld V, Habermann K, Dolgikh N, Fulda S. Targeting ferroptosis in rhabdomyosarcoma cells. Int J Cancer. 2020;146:510–20. 31173656 10.1002/ijc.32496

[15] Ding H, Chen S, Pan X, Dai X, Pan G, Li Z, et al. Transferrin receptor 1 ablation in satellite cells impedes skeletal muscle regeneration through activation of ferroptosis. J Cachexia Sarcopenia Muscle. 2021;12:746–68. 33955709 10.1002/jcsm.12700 PMC8200440

[16] Wei L, Li Y, Tan H, Peng Y, Liu Q, Zheng T, et al. OTUB1 regulates ferroptosis to inhibit myoblast differentiation into myotubes by deubiquitinating P62. Sci Rep. 2024;14:15696. 38977909 10.1038/s41598-024-66868-3 PMC11231240

[17] Wang Y, Zhang Z, Jiao W, Wang Y, Wang X, Zhao Y, et al. Ferroptosis and its role in skeletal muscle diseases. Front Mol Biosci. 2022;9:1051866. 36406272 10.3389/fmolb.2022.1051866 PMC9669482

[18] Xiao R, Wei Y, Zhang Y, Xu F, Ma C, Gong Q, et al. Trilobatin, a naturally occurring Food Additive, ameliorates exhaustive Exercise-Induced fatigue in mice: involvement of Nrf2/ARE/Ferroptosis Signaling Pathway. Front Pharmacol. 2022;13:913367. 35814232 10.3389/fphar.2022.913367 PMC9263197

[19] Yang H, Li Y, Zhu W, Feng X, Xin H, Chen H, et al. SAT1/ALOX15 signaling pathway is involved in ferroptosis after skeletal muscle contusion. Int J Mol Sci. 2024;25:11317. 39457099 10.3390/ijms252011317 PMC11508450

[20] Lin Y, Luo T, Weng A, Huang X, Yao Y, Fu Z, et al. Gallic acid alleviates gouty arthritis by inhibiting NLRP3 inflammasome activation and pyroptosis through enhancing Nrf2 signaling. Front Immunol. 2020;11:580593. 33365024 10.3389/fimmu.2020.580593 PMC7750458

[21] Govea-Salas M, Rivas-Estilla AM, Rodríguez-Herrera R, Lozano-Sepúlveda SA, Aguilar-Gonzalez CN, Zugasti-Cruz A, et al. Gallic acid decreases Hepatitis C virus expression through its antioxidant capacity. Exp Ther Med. 2016;11:619–24. 26893656 10.3892/etm.2015.2923 PMC4734044

[22] Fanaei H, Mard SA, Sarkaki A, Goudarzi G, Khorsandi L. Gallic acid treats dust-induced NAFLD in rats by improving the liver’s anti-oxidant capacity and inhibiting ROS/NFκβ/TNFα inflammatory pathway. Iran J Basic Med Sci. 2021;24:240–7. 33953864 10.22038/IJBMS.2021.51036.11603 PMC8061332

[23] Kosuru RY, Roy A, Das SK, Bera S. Gallic acid and gallates in Human Health and Disease: do Mitochondria hold the Key to Success? Mol Nutr Food Res. 2018;62. 10.1002/mnfr.201700699 29178387

[24] Xie J, Wang H, Xie W, Liu Y, Chen Y. Gallic acid promotes ferroptosis in hepatocellular carcinoma via inactivating Wnt/β-catenin signaling pathway. Naunyn Schmiedebergs Arch Pharmacol. 2024;397:2437–45. 37847411 10.1007/s00210-023-02770-5

[25] Li Z, Liu S-Y, Xu L, Xu S-Y, Ni G-X. Effects of treadmill running with different intensity on rat subchondral bone. Sci Rep. 2017;7:1977. 28512292 10.1038/s41598-017-02126-z PMC5434052

[26] Yan K, Gao H, Liu X, Zhao Z, Gao B, Zhang L. Establishment and identification of an animal model of long-term exercise-induced fatigue. Front Endocrinol (Lausanne). 2022;13:915937. 36093084 10.3389/fendo.2022.915937 PMC9459130

[27] Dixon SJ, Lemberg KM, Lamprecht MR, Skouta R, Zaitsev EM, Gleason CE, et al. Ferroptosis: an iron-dependent form of nonapoptotic cell death. Cell. 2012;149:1060–72. 22632970 10.1016/j.cell.2012.03.042 PMC3367386

[28] Helli B, Navabi SP, Hosseini SA, Sabahi A, Khorsandi L, Amirrajab N et al. The Protective effects of Syringic Acid on Bisphenol A-Induced Neurotoxicity possibly through AMPK/PGC-1α/Fndc5 and CREB/BDNF signaling pathways. Mol Neurobiol. 2024 Oct;61(10):7767-7784. 10.1007/s12035-024-04048-0 38430353

[29] Bai Y, Li K, Li X, Chen X, Zheng J, Wu F, et al. Effects of oxidative stress on hepatic encephalopathy pathogenesis in mice. Nat Commun. 2023;14:4456. 37488119 10.1038/s41467-023-40081-8 PMC10366183

[30] Rendon CJ, Flood E, Thompson JM, Chirivi M, Watts SW, Contreras GA. PIEZO1 mechanoreceptor activation reduces adipogenesis in perivascular adipose tissue preadipocytes. Front Endocrinol (Lausanne). 2022;13:995499. 36120469 10.3389/fendo.2022.995499 PMC9471253

[31] Yang R, Shi L, Si H, Hu Z, Zou L, Li L, et al. Gallic Acid Improves Comorbid Chronic Pain and Depression Behaviors by Inhibiting P2X7 Receptor-Mediated Ferroptosis in the Spinal Cord of Rats. ACS Chem Neurosci. 2023;14:667–76. 36719132 10.1021/acschemneuro.2c00532

[32] Macedo-Dias JA, Queiróz Machado F, Vouga L, Gonçalves V, Gomes R. Liposarcoma of the heart. A case report. Am J Cardiovasc Pathol. 1990;3:259–63. 2095832

[33] M L-F JN, Sh AR et al. F, B L, Y A,. Skeletal muscle necrosis and regeneration after injection of Thalassophryne nattereri (niquim) fish venom in mice. International journal of experimental pathology [Internet]. 2001 [cited 2024 Sep 19];82. Available from: https://pubmed.ncbi.nlm.nih.gov/11422541/10.1046/j.1365-2613.2001.00181.x PMC2517697 11422541

[34] Sudhakar S, Kirthika SV. Enhancing skeletal muscle Rehabilitation: the effects of Diclofenac Phonophoresis and Shock Wave Therapy on serum creatine kinase in athletes with delayed-onset muscle soreness. Cureus. 2023;15:e46267. 37908915 10.7759/cureus.46267 PMC10615225

[35] T I, H I, N N SY et al. M M, A M,. Bioimpedance analysis for identifying new indicators of exercise-induced muscle damage. Scientific reports [Internet]. 2024 [cited 2024 Nov 21];14. Available from: https://pubmed.ncbi.nlm.nih.gov/38961243/10.1038/s41598-024-66089-8 PMC11222495 38961243

[36] Washington TA, Reecy JM, Thompson RW, Lowe LL, McClung JM, Carson JA. Lactate dehydrogenase expression at the onset of altered loading in rat soleus muscle. J Appl Physiol (1985). 2004;97:1424–30. 10.1152/japplphysiol.00222.2004 15358753

[37] Wang Z-Z, Xu H-C, Zhou H-X, Zhang C-K, Li B-M, He J-H, et al. Long-term detraining reverses the improvement of lifelong exercise on skeletal muscle ferroptosis and inflammation in aging rats: fiber-type dependence of the Keap1/Nrf2 pathway. Biogerontology. 2023;24:753–69. 37289374 10.1007/s10522-023-10042-1

[38] Alves FM, Ayton S, Bush AI, Lynch GS, Koopman R. Age-related changes in skeletal muscle Iron homeostasis. J Gerontol Biol Sci Med Sci. 2023;78:16–24. 10.1093/gerona/glac139 35869751

[39] Wang Y, Yan S, Liu X, Deng F, Wang P, Yang L, et al. PRMT4 promotes ferroptosis to aggravate doxorubicin-induced cardiomyopathy via inhibition of the Nrf2/GPX4 pathway. Cell Death Differ. 2022;29:1982–95. 35383293 10.1038/s41418-022-00990-5 PMC9525272

[40] Fang Y, Chen X, Tan Q, Zhou H, Xu J, Gu Q. Inhibiting ferroptosis through disrupting the NCOA4-FTH1 Interaction: a new mechanism of action. ACS Cent Sci. 2021;7:980–9. 34235259 10.1021/acscentsci.0c01592 PMC8227600

[41] Gao M, Jiang X. To eat or not to eat-the metabolic flavor of ferroptosis. Curr Opin Cell Biol. 2018;51:58–64. 29175614 10.1016/j.ceb.2017.11.001 PMC5949249

[42] Li H, Horke S, Förstermann U. Oxidative stress in vascular disease and its pharmacological prevention. Trends Pharmacol Sci. 2013;34:313–9. 23608227 10.1016/j.tips.2013.03.007

[43] Liu H, Zhou L, Wang X, Zheng Q, Zhan F, Zhou L, et al. Dexamethasone upregulates macrophage PIEZO1 via SGK1, suppressing inflammation and increasing ROS and apoptosis. Biochem Pharmacol. 2024;222:116050. 38354960 10.1016/j.bcp.2024.116050

[44] Ar MS, Rm N, At L. C. The Form and Function of PIEZO2. Annual review of biochemistry [Internet]. 2021 [cited 2024 Sep 19];90. Available from: https://pubmed.ncbi.nlm.nih.gov/34153212/10.1146/annurev-biochem-081720-023244 PMC8794004 34153212

[45] Mohamed EK, Hafez DM. Gallic acid and metformin co-administration reduce oxidative stress, apoptosis and inflammation via Fas/caspase-3 and NF-κB signaling pathways in thioacetamide-induced acute hepatic encephalopathy in rats. BMC Complement Med Ther. 2023;23:265. 37491245 10.1186/s12906-023-04067-9 PMC10367384

