# Supplementary material for: Gallic acid alleviates exercise-induced muscle damage by inhibiting mitochondrial oxidative stress and ferroptosis
Source: J Transl Med. 2025 Jan 8;23:30. doi: 10.1186/s12967-024-06042-5 (PMC11707880; doi:10.1186/s12967-024-06042-5)
Supplement: Supplementary file 1 — Supplementary Material 1 [file 12967_2024_6042_MOESM1_ESM.docx]

**
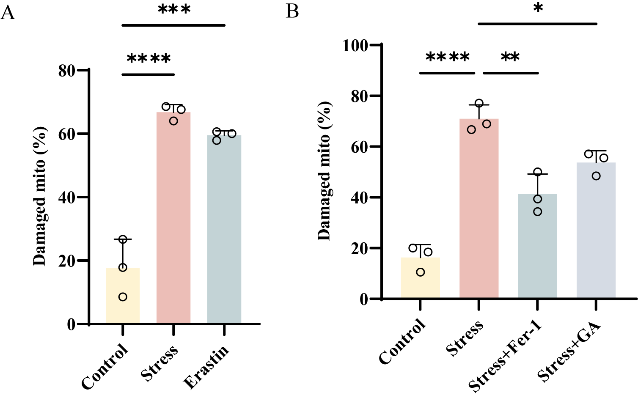
Fig. S1 Quantitative analysis of mitochondria using TEM**

A．The quantitative analysis bar chart of damaged mitochondria under TEM represented by Figure 3H.

B．The quantitative analysis bar chart of damaged mitochondria under TEM represented by Figure 4G.

Values are expressed as the mean ± SD from three independent experiments (n = 3). **p* < 0.05, ***p* < 0.01, ****p* < 0.001, *****p* < 0.0001.

**
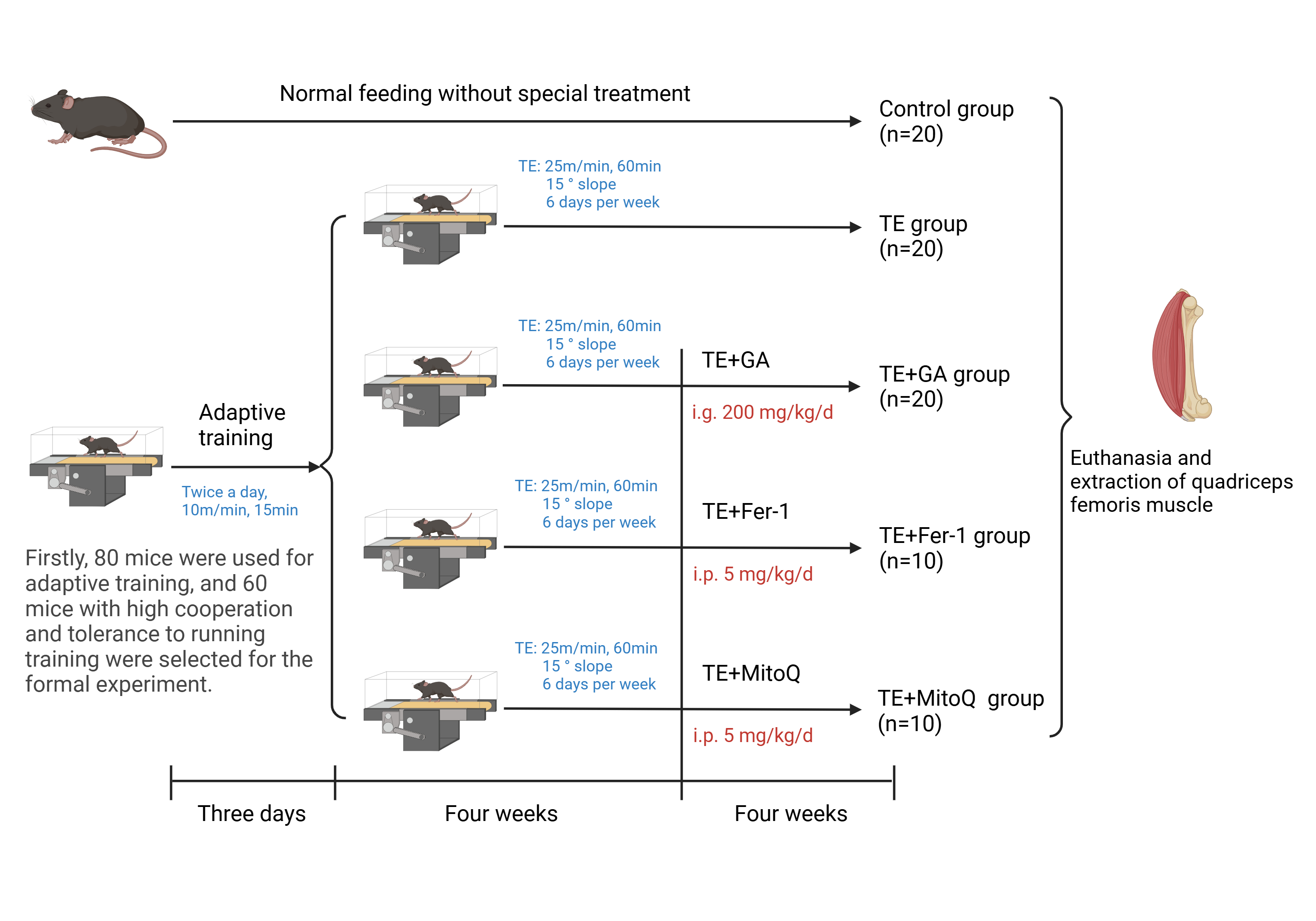
Fig. S2 Mice treatment protocol**


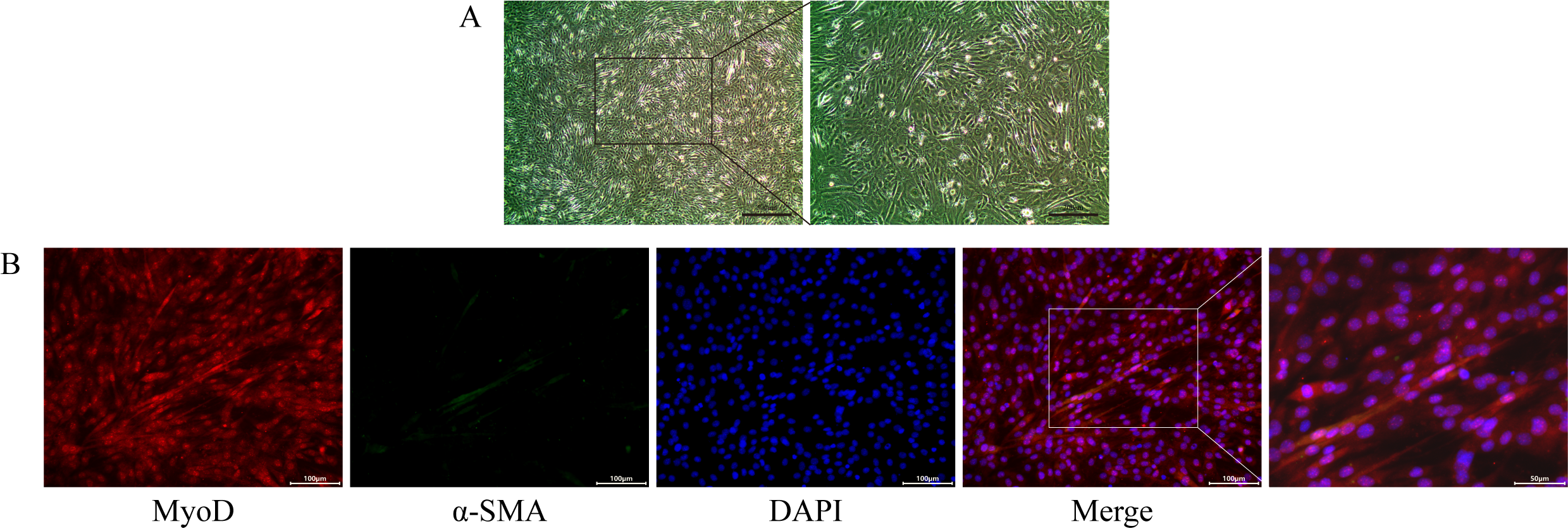


**Fig. S3 Identification of skeletal muscle cells**

A. Observation of skeletal muscle cells under inverted microscope, where "_" represents 500 µm.

B. Immunofluorescence detection of MyoD and α-SMA expression in skeletal muscle cells, where "_" represents 100 µm.


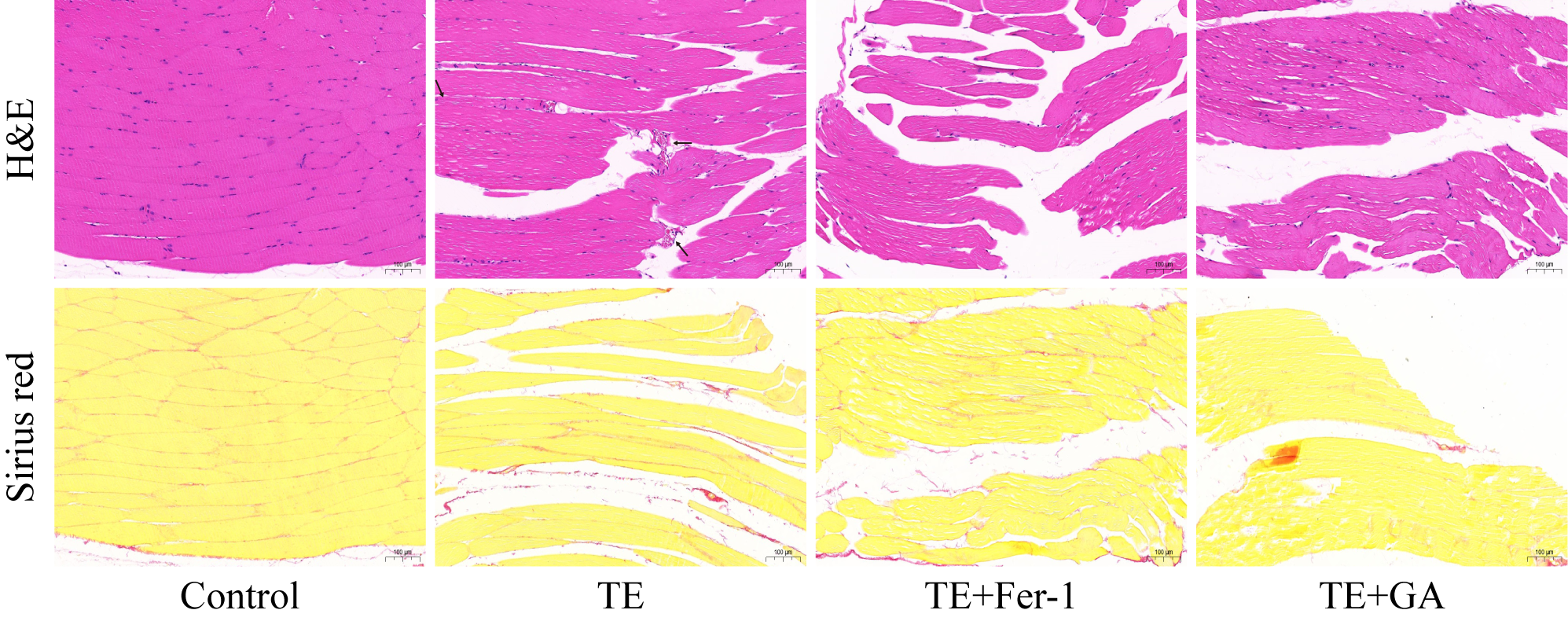


**Fig. S4 Images of mouse skeletal muscle stained with H&E and Sirius red**

**
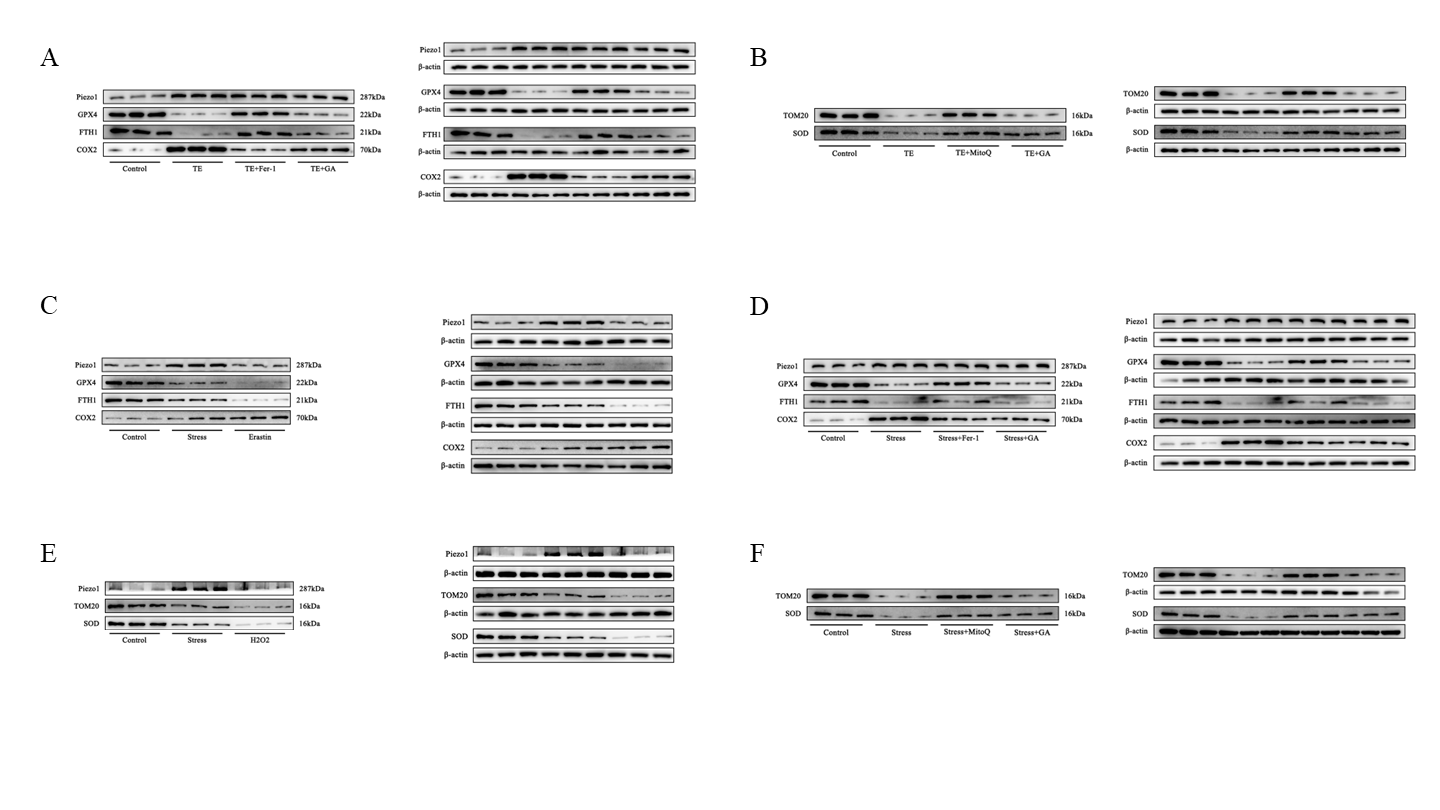
**

**Fig. S5 Western blot images in each figure**

A. Fig. 1D corresponding Western blot image

B. Fig. 2D corresponding Western blot image

C. Fig. 3I corresponding Western blot image

D. Fig. 4H corresponding Western blot image

E. Fig. 5J corresponding Western blot image

F. Fig. 6F corresponding Western blot image

**TableS1 List of Abbreviations**

| **Abbreviation** | **Full Term** |
| --- | --- |
| EIMD | exercise-induced muscle damage |
| GA | Gallic acid |
| TE | Treadmill Exercise |
| CK | creatine kinase |
| LDH | lactate dehydrogenase |
| IL | interleukin |
| TNF-α | tumour necrosis factor-alpha |
| ROS | reactive oxygen species |
| GPX4 | Glutathione peroxide reductase |
| GSH | glutathione |
| Fer-1 | Ferrostatin-1 |
| Mito Q | Mitoquinone |
| MDA | Malondialdehyde |
| COX2 | cyclooxygenase-2 |
| GSSG | Glutathione oxidized |
| SOD | superoxide dismutase |
| FTH1 | Ferritin Heavy Chain 1 |
| Fe^2+^ | ferrous iron |
| DAMP | damage-associated molecular patterns |
