# Supplementary material for: Gallic acid alleviates exercise-induced muscle damage by inhibiting mitochondrial oxidative stress and ferroptosis
Source: J Transl Med. 2025 Jan 8;23:30. doi: 10.1186/s12967-024-06042-5 (PMC11707880; doi:10.1186/s12967-024-06042-5)
Supplement: Supplementary file 2 — Supplementary Material 2 [file 12967_2024_6042_MOESM2_ESM.docx]

**Response Letter**

Ref: No. JTRM-D-24-12008. “Gallic acid alleviates exercise-induced muscle damage by inhibiting mitochondrial oxidative stress and ferroptosis ” by Likai Yu et al.

Dec. 15, 2024

Dear editor and reviewers,

On behalf of my co-authors, we are very grateful for your constructive comments and suggestions for our manuscript. Your comments are very valuable and helpful for improving our manuscript.

In the following, the responses to all the comments are provided one by one. The revised part is marked in red font in the manuscript. Comments are in black and numbered. Our responses are in red, and quoted texts from the manuscript are in blue.

Based on the reviewer's comments, we have made revisions to the manuscript and added additional data to make the results more convincing. Thank you again to the editors and reviewers for your patient work and valuable suggestions, which have important guiding significance for our research.

Sincerely,

Corresponding author

Nongshan Zhang,

Jiangsu Province Hospital of Chinese Medicine,

Nanjing, Jiangsu, China

E-mail: zns0032@163.com

Reviewer #2:

Thank you for your strong support of our work, you have provided us with very valuable advice to improve the quality of this paper! We have used your comments to revise the manuscript and have attached a point-by-point response to your comments. Comments are in black and numbered. Our responses are in red, and quoted texts from the manuscript are in blue.

1. For response to comment 2:

The authors must clearly state how many mice were initially selected for treadmill training and include this number in the Fig. S2 Protocol. Numbers such as 20 and 10 seem unrealistic. Additionally, if the authors initially captured both longitudinal and cross-sectional views, they must show both and include one of them in the supplementary figures. Finally, there are still no inflammatory cells clearly visible in the HE staining images, which casts doubt on the claims of inflammation and treadmill-induced injury. The authors must provide an intersect image with arrows clearly indicating the inflammatory cells in the HE staining.

Thank you for your feedback. We have carefully reviewed the original manuscript and made revisions according to your comments. Firstly, we initially selected 80 mice for screening. Based on the experimental requirements, we selected 60 mice with high cooperation and tolerance to running training through early adaptive training to be included in the formal experiment. As shown in the following figure, we have added specific numbers and filtering requirements to Fig.S2 based on your comments。

Fig. S2 Mice treatment protocol

In addition, the treadmill equipment we selected can set the movement speed and time, and a micro electrical stimulation device is installed behind the treadmill area of this equipment, which can make the mice run continuously without harming the animals. Researchers are also present to observe the mice's movement throughout the experiment to ensure the smooth completion of the experiment. The following image shows a mouse undergoing treadmill training.

Finally, we added HE stained images of longitudinal muscle tissue sections in the supplementary data. In Fig S4 and Fig. 1A, we have added arrows to indicate the location of inflammatory infiltration in the tissue, as shown in the following figure. We appreciate for your warm work earnestly and hope that the correction will meet with approval.

Fig. S4 Images of mouse skeletal muscle stained with H&E and Sirius red

Fig. 1A Images of mouse skeletal muscle stained with H&E and Sirius red, “_” represents 100 µm.

2. The response to comment 4:

This response is not convincing. Identifying the source of inflammatory cytokines is critical to this study, as it would clarify the GA-targeted cell type. Alleviating oxidative stress in muscle cells may not be the main mechanism of GA. Particularly, the authors claim that treadmill-induced muscle injury attracts inflammatory cell infiltration (as it typically does). Theoretically, inflammatory cells such as neutrophils and monocytes should secrete significantly higher levels of inflammatory cytokines compared to muscle cells.

Thank you very much for your professional comments on our article, pointing out our shortcomings and providing great help for improving our future research on this disease and drugs. The theoretical basis of our research is that vigorous exercise stimulates the active muscle fibers in muscles to produce a large amount of reactive oxygen species (ROS), which leads to extensive oxidative damage and inflammatory reactions, accelerating muscle fatigue in the body. At the same time, literature suggests that skeletal muscle is the main source of ROS production during exercise. (*Powers SK, Deminice R, Ozdemir M, et al. Exercise-induced oxidative stress: Friend or foe? J Sport Health Sci. 2020 Sep;9(5):415-425.*) Therefore, our research mainly focuses on skeletal muscle cells. The results also demonstrate that gallic acid (GA) can effectively intervene in the oxidative stress response and ferroptosis of skeletal muscle cells, which is also the focus of this study. We believe that if GA can intervene in the ROS produced by skeletal muscle cells and subsequent oxidative stress and iron death events, it can greatly alleviate muscle damage caused by intense exercise. After receiving your comment, we realized that as GA has been proven to have a wide range of anti-inflammatory effects, it seems impossible to prove the source of the inflammatory factors we intervened in based on ELISA experiments. GA may indirectly have a therapeutic effect on skeletal muscle by intervening in systemic inflammatory responses. We have noted this research deficiency in the manuscript (Line 486), “In addition, due to the widely proven anti-inflammatory effects of GA, our study only indicates that the ferroptosis and oxidative stress response of skeletal muscle cells caused by vigorous exercise can be effectively alleviated by GA. But the recovery of skeletal muscle inflammation injury may be indirectly affected by its systemic anti-inflammatory effect.” Thank you for pointing out the clear direction for our next research. We plan to identify the specific mechanism of skeletal muscle inflammation caused by intense exercise and the main therapeutic effect of GA in our next experimental study.

3. The response to comment 5:

If the authors have already performed same-membrane normalization, they should include these in the representative figures, such as displaying the target proteins along with the same membrane actin.

Thank you sincerely for your patient work and attention to the rigor of our experiment. As shown in the figure below, we have provided target proteins and their corresponding internal reference images in the supplementary data. Thank you again for your comments and valuable suggestions, which have greatly helped improve the quality of our manuscript.

**Fig. S5 Western blot images in each figure**

A. Fig. 1D corresponding Western blot image

B. Fig. 2D corresponding Western blot image

C. Fig. 3I corresponding Western blot image

D. Fig. 4H corresponding Western blot image

E. Fig. 5J corresponding Western blot image

F. Fig. 6F corresponding Western blot image
